# Synthesis of (–)-melazolide B, a degraded limonoid, from a natural terpene precursor^☆^

**DOI:** 10.1016/j.tchem.2022.100011

**Published:** 2022-03-22

**Authors:** Yannan Liu, Alexander W. Schuppe, Yizhou Zhao, Jaehoo Lee, Timothy R. Newhouse

**Affiliations:** Department of Chemistry, Yale University, 225 Prospect Street, New Haven, CT, 06520-8107, United States

**Keywords:** Melazolide B, Limonoid, Total synthesis, Natural product, Terpenoid

## Abstract

Degraded limonoids are a subclass of limonoid natural products that derive from ring-intact or ring-rearranged limonoids. Establishment of robust synthetic routes to access them could provide valuable materials to identify the simplest active pharmacophore responsible for the observed biological activities of the parent molecules. This communication delineates the development of a divergent strategy to furnish melazolide B and several other related congeners from a common keto-lactone intermediate, which was rapidly assembled from α-ionone. A chemoselective carbonyl α,β-dehydrogenation and a Wharton reduction were key strategic steps in this synthetic pathway.

## 1. Introduction

**Scheme 1.**
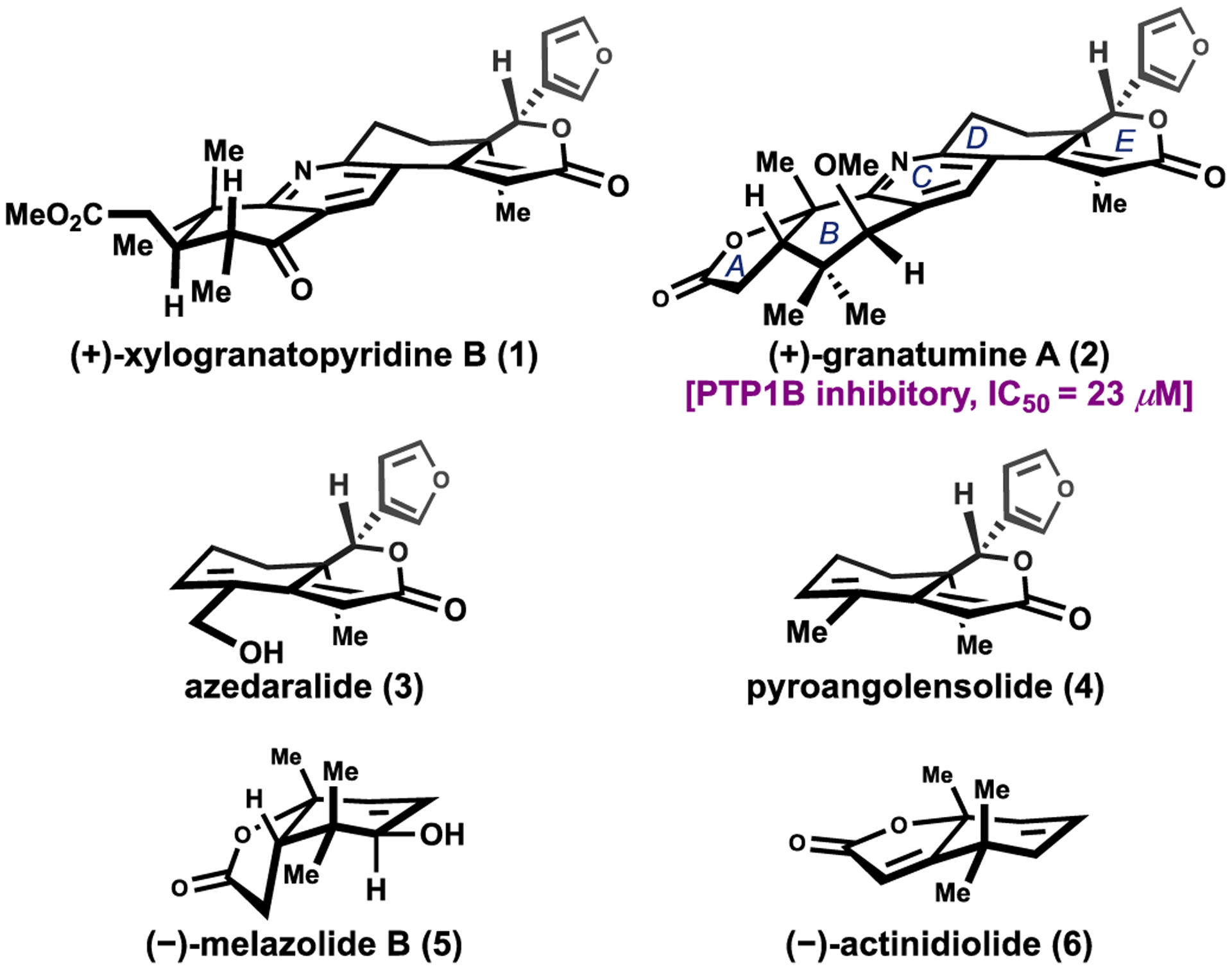
Selected limonoids and structurally related compounds.

Limonoids are a large family of terpenoid natural products with more than a thousand members isolated to date [1–3]. These secondary metabolites display a broad array of biological activities, ranging from anticancer, anti-inflammation, antifeedant, and neurological activities [1–3]. Due to their diverse and intricate structures as well as interesting biological profiles, several synthetic campaigns have targeted this family of natural products [4–13]. Our group has reported the total synthesis of several rearranged limonoids and pyridine-containing bislactone limonoid alkaloids (**1–2**) [10–12], which exhibit modest PTP1B inhibitory activity (Scheme 1) [14]. The development of these robust synthetic routes has enabled efficient access to them and their analogs for SAR studies [15]. These investigations piqued our interest in identifying the active pharmacophore for the observed PTP1B inhibition.

**Scheme 2.**
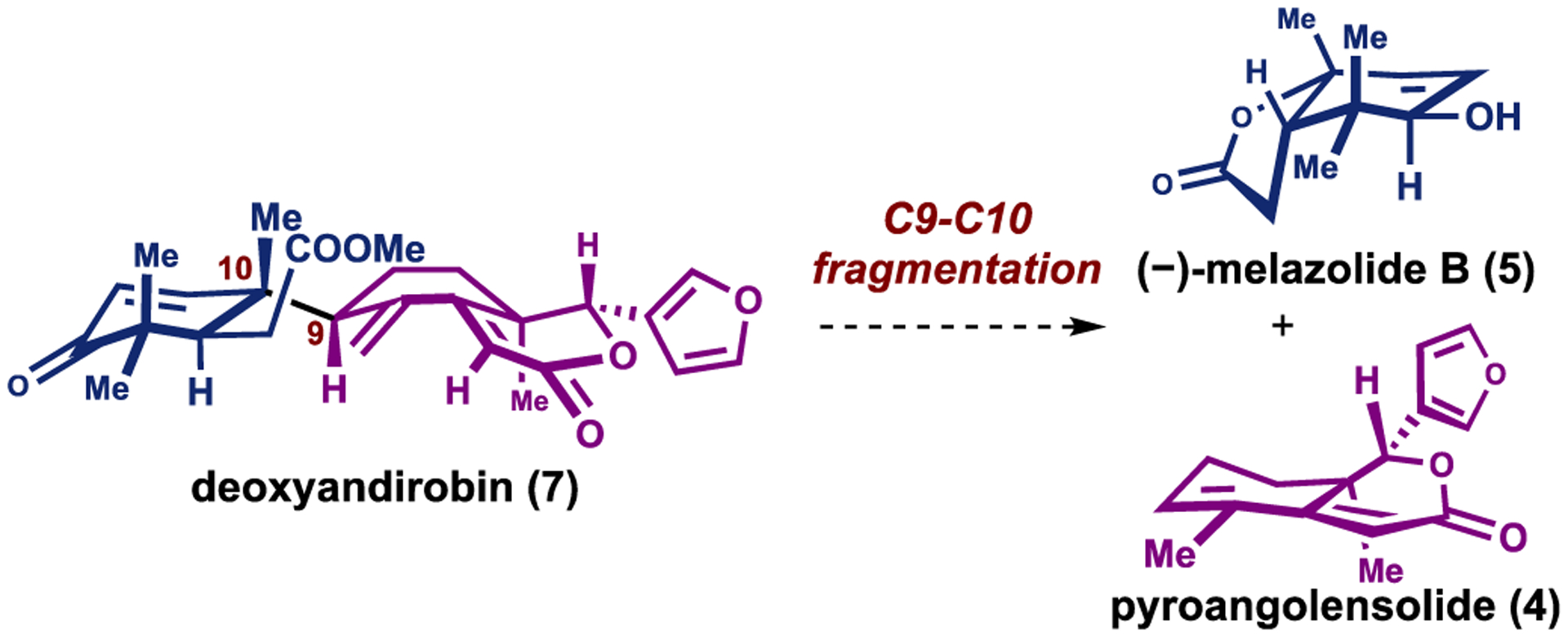
Proposed biosynthesis of (–)-melazolide B.

Degraded limonoids, such as azedaralide (**3**), pyroangolensolide (**4**) and (–)-melazolide B (**5**), are a subclass of limonoid natural products arising from ring-intact or ring-rearranged limonoids (Scheme 1) [16]. Although highly speculative at this juncture, Guerriero and co-workers hypothesized, based on the co-isolation of pyroangolensolide (**4**) and melazolide B (**5**), that they may derive from a common tetranotriterpenoid precursor such as deoxyandirobin (**7**) (Scheme 2) [16].Fragmentation along the C9–C10 bond would yield fragments resembling degraded limonoid natural products (Scheme 2) [16]. Mechanistic proposals have previously been hypothesized [17,18].

Our established approach provided a synthetic pathway to access compounds related to the DE-ring fragments, such as azedaralide (**3**) and pyroangolensolide (**4**) [12]. Herein we disclose the development of synthetic routes to access compounds related to the AB-ring fragments, including (–)-melazolide B (**5**) and actinidiolide (**6**).

## 2. Results and discussion

**Scheme 3.**
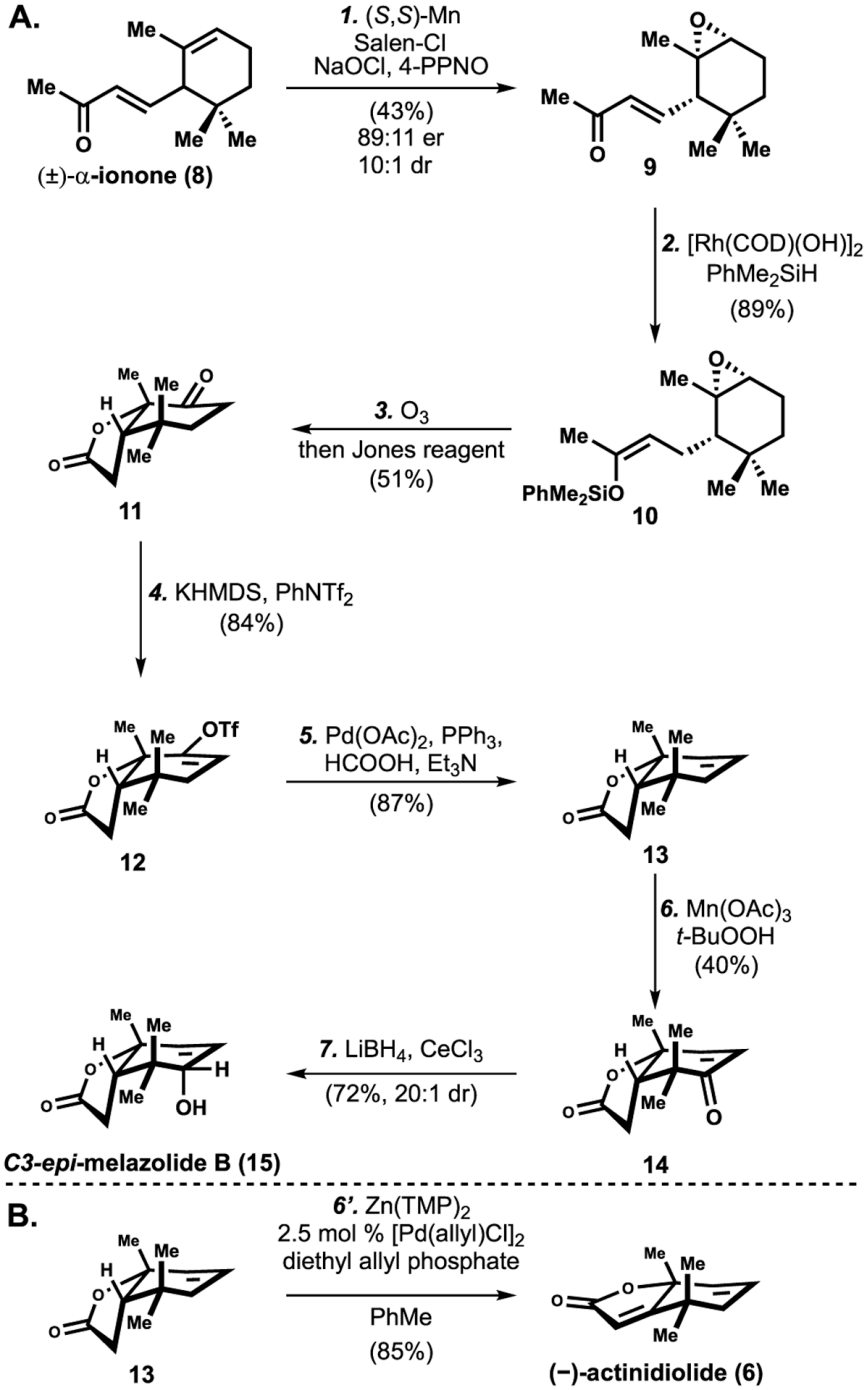
Synthesis of C3-*epi*-melazolide B^a^. ^*a*^Reagents and conditions: (1) 5 mol % (*S,S*)-(+)-*N,N*′-Bis(3,5-di-*tert*-butylsali-cylidene)-1,2-cyclohexanediaminomanganese(III) chloride, 4-phenylpyridine-*N*-oxide (5 mol %), aq. NaOCl (1 equiv), CH_2_Cl_2_, 0 to 23 °C, 43%, 89:11 er, 10:1 dr; (2) 1 mol % [Rh(COD)(OH)]_2_, PhMe_2_SiH (1.3 equiv), THF, 23 to 60 °C, 2 h, 89%; (3) O_3_, acetone, 78 °C, 0.5 h, then Jones reagent (2.0 equiv), 0 to 23 °C, 2 h, 51%; (4) KHMDS (1.3 equiv), PhNTf_2_ (1.3 equiv), THF, −78 to 23 °C, 1 h, 84%; (5) 5 mol % Pd(OAc)_2_, 10 mol % PPh_3_, formic acid (2.0 equiv), Et_3_N (3.0 equiv), DMF, 60 °C, 0.5 h, 87% (6) Mn(OAc)_3_ (0.7 equiv), TBHP (5.0 equiv), EtOAc, 70 °C, 3 d, 40%; (7) LiBH_4_ (2.0 equiv), CeCl_3_ (2.0 equiv), THF/MeOH, 0 °C to rt, 1 h, 72%, 20:1 dr; (6′) Zn(TMP)_2_ (1.0 equiv), diethyl allyl phosphate (1.0 equiv), 2.5 mol % [Pd(allyl)Cl]_2_, PhMe, 120 °C, 3 h, 85%.

Our initial synthetic strategy focused on a bidirectional search between the known degraded limonoids, such as **5** and **6**, and our previously reported intermediate **11**. The benefit of **11** as a starting material goal is that it already contains the key ring systems, quaternary center, and two stereocenters common to these degraded limonoids [19]. A cyclohexanone would need to be converted to an allylic alcohol wherein the hydroxyl group has formally undergone a reductive transposition. As an added benefit, the route to **11** was robust, and involved conversion of α-ionone (**8**) by a three-step sequence involving a kinetic resolution via Jacobsen epoxidation [20], 1,4-hydrosilylation, and oxidative cleavage, as shown in Scheme 3A [12]. Treatment of ketone **11** with KHMDS and *N*-phenyl-bis(trifluoromethanesulfonimide) resulted in the formation of an intermediate vinyl triflate (**12**) in 84% yield, which was then reduced to alkene **13** in 87% yield (Scheme 3A). Other reductants employed in this Pd-catalyzed reduction, including Et_3_SiH and Bu_3_SnH, were less effective in this context.

Conversion of alkene **13** to an intermediate enone was achieved by allylic oxidation utilizing Mn(OAc)_3_ and t-BuOOH [21]. Employing alternative allylic oxidation conditions to furnish **5** or **15** directly, such as SeO_2_ and Cr-based oxidants, were unsuccessful. A diastereoselective Luche reduction of the enone intermediate (**14**) resulted exclusively in the formation of C3-epi-melazolide B (**15**).

Although **13** was not a viable intermediate to melazolide B (**5**), considering our laboratory’s lactone α,β-dehydrogenation [22], we reasoned that subjection of **13** to lactone α,β-dehydrogenation conditions could give rise to (–)-actinidiolide (**6**), an ionone-related compound that was proposed to be produced from kiwiionoside in nature [23]. Indeed, dehydrogenation of the lactone functionality in **13** with our laboratory’s allyl Pd-catalyzed dehydrogenation conditions revealed that the conditions originally developed for ketone dehydrogenation were most effective (the Zn(TMP)_2_ and diethyl allyl phosphate system) to produce actinidiolide (**6**), as shown in scheme 3B [22]. Employing the conditions previously developed by our laboratory for ester [24] or amide [25] dehydrogenation resulted in lower conversion and diminished yield (23% and 47% yield respectively). These results suggest that the Zn(TMP)_2_ system may be more general for the dehydrogenation of other basic functionalities.

**Scheme 4.**
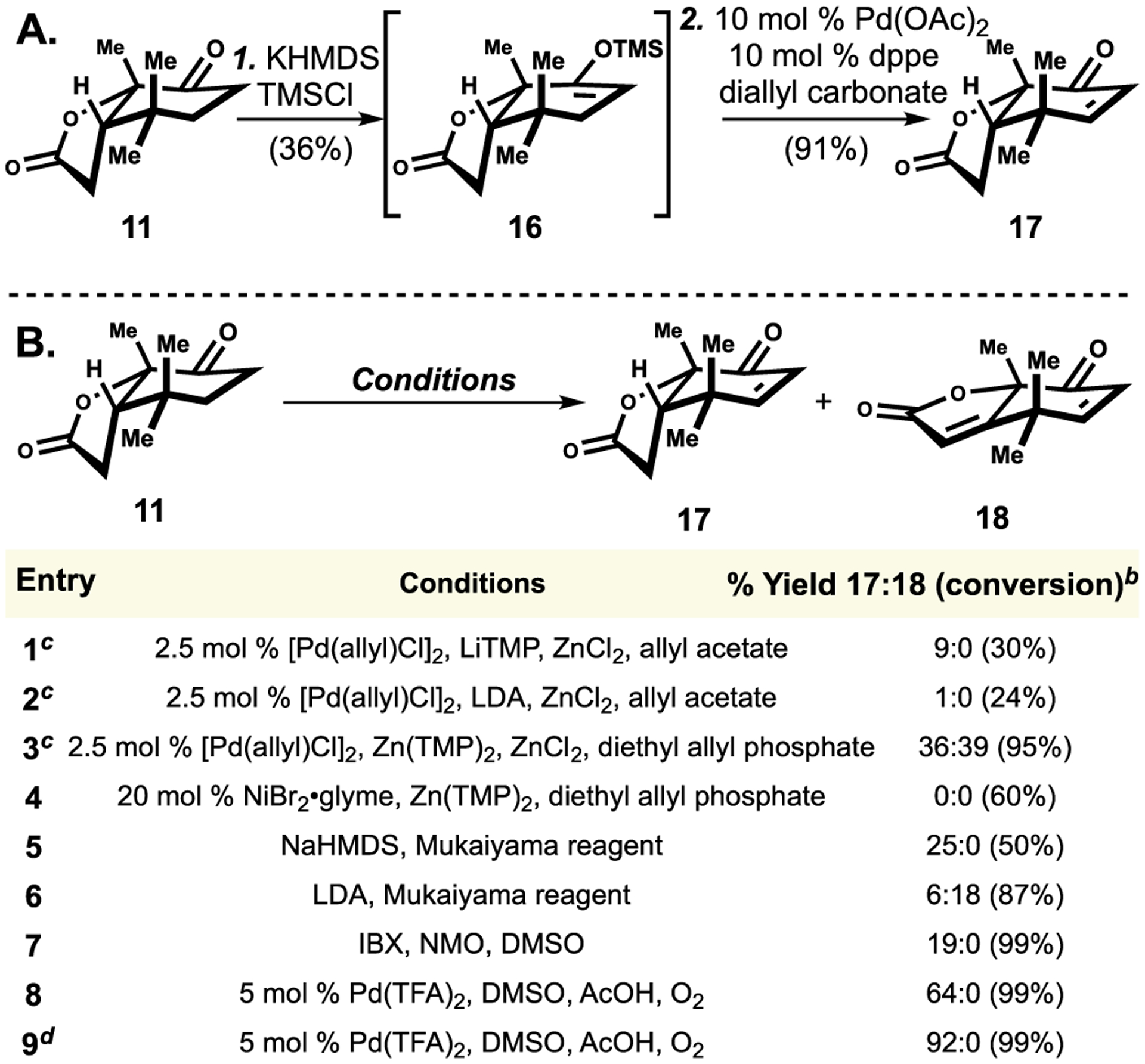
Optimization of ketone dehydrogenation^a^. ^*a*^Reagents and conditions: (1) KHMDS (1 equiv), TMSCl (1.5 equiv), THF, −78 °C to rt, 2 h 36%; (2) 10 mol % Pd(OAc)_2_, 10 mol % dppe, diallyl carbonate (1.5 equiv), MeCN, 80 °C, 4 h, 91%. ^*b*^Yield of the crude reaction mixture, using 0.05 mmol **11**, was determined by ^1^H NMR using dibromomethane as an internal standard. Conversion of **11** in parenthesis. ^*c*^Reactions conducted on 0.2 mmol scale. ^*d*^Conducted on a 3-gram scale.

In order to obtain melazolide B (**5**), we undertook an alternative route through enone **17** (Scheme 4). Several ketone dehydrogenation conditions of **11** was first examined. A two-step sequence involving TMS enol ether formation and dehydrogenation was first attempted. Treatment of ketone **11** with KHMDS and TMSCl resulted in **16** in 36% yield (Scheme 4A). Although the original Saegusa-Ito oxidation conditions only led to full decomposition of the sily enol ether starting material (**16**) [26], subjection of **16** to Tsuji’s modified conditions smoothly delivered the enone product (**17**) in 91% yield (Scheme 4A) [27].

Several one-step dehydrogenation conditions were surveyed (Scheme 4B). Utilizing our laboratory’s allyl-Pd-catalyzed dehydrogenation conditions [22] (entries 1–3) resulted in overoxidation (**18**), however, the Ni-catalyzed dehydrogenation conditions only gave partially recovered starting material (entry 4). Employing either Mukaiyama’s reagent [28] (entries 5–6) or IBX (entry 7) resulted in minimal desired product [29]. Subjection of **11** to Stahl’s Pd-catalyzed aerobic dehydrogenation conditions afforded enone **17** with excellent selectivity (>20:1, entry 8) and upon conducting on a 3-g scale, excellent yield (92% yield, entry 9) [30].

**Scheme 5.**
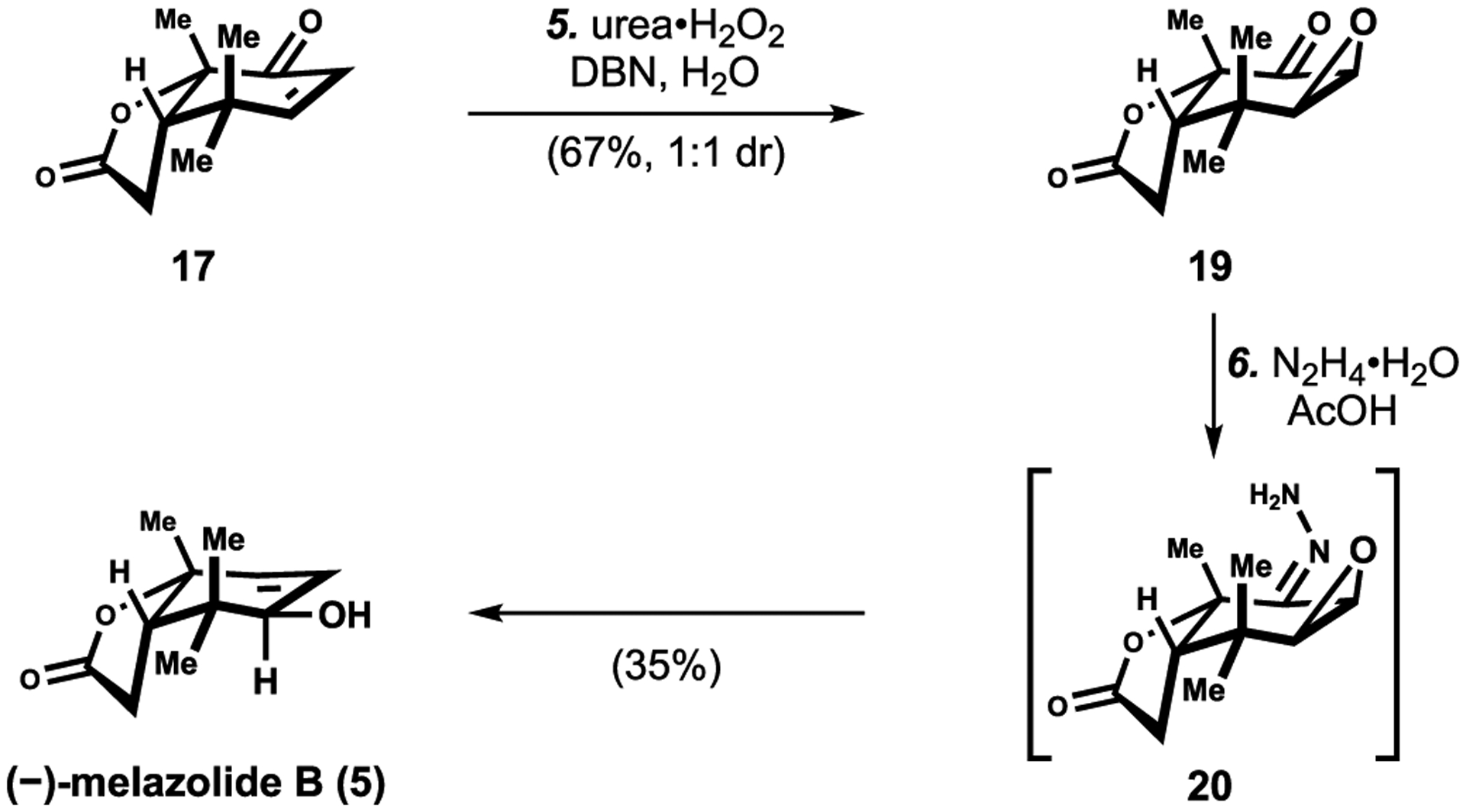
Synthesis of (–)-melazolide B through Wharton reduction^a^. ^*a*^Reagents and conditions: (5) Urea⋅H_2_O_2_ (3.0 equiv), DBN (3.0 equiv), H_2_O (9.0 equiv), THF, 0 to 23 °C, 5 h, 67%, 1:1 dr; (6) N_2_H_4_⋅H_2_O (3.0 equiv), AcOH (1.5 equiv), MeOH, rt, 24 h, 35%.

With enone **17** in hand, we were ready to test the proposed synthesis of melazolide B (**5**) through Wharton reduction [31]. Treatment of enone **17** with urea∙H_2_O_2_ effected a nucleophilic epoxidation to furnish epoxide 19 in 67% yield as a 1:1 mixture of diastereomers (Scheme 5) [12]. Reduction of the mixture of diastereomers of the α,β-epoxy ketone (**19** with Wharton’s hydrazine protocol resulted in the formation of (–)-melazolide B (5), via the intermediacy of hydrazone **20** (Scheme 5) [31]. Interestingly, during the reduction of the α,β-epoxy ketone (**19**) with hydrazine only the desired diastereomer of the allylic alcohol **5** was observed, whereas the undesired diastereomer was degraded. It is unclear at this juncture what those decomposition pathway or pathways are, however the presence of an electrophilic lactone was possibly a liability. This six-step sequence marks the first reported synthesis of (–)-melazolide B.

## 3. Conclusion

In summary, we have documented efficient synthesis of C3-*epi*-melazolide B, melazolide B and actinidiolide. Tapping into the natural terpene precursors by utilizing α-ionone as a starting material accelerated the assembly of the core bicyclic structures and led to the first reported synthesis of (–)-melazolide B. Among the reported synthesis of (–)-actinidiolide (**6**) including those from Jorgensen (7 steps) [32], Eidman (7 steps) [33], and Mori (10 steps) [34–36], this six-step sequence represents an alternative and concise asymmetric synthesis of (–)-actinidiolide (**6**). Our robust and scalable pathway will enable future investigations into the PTP1B inhibition of these and similar compounds.

## Supplementary Material

Supplemental Informatio
