# Supplementary material for: Synthesis of (–)-melazolide B, a degraded limonoid, from a natural terpene precursor
Source: Tetrahedron Chem. Author manuscript; Available in PMC 2024 Apr 11. (PMC11008529; doi:10.1016/j.tchem.2022.100011)
Supplement: Supplemental Informatio [file NIHMS1977277-supplement-Supplemental_Informatio.pdf]

Synthesis of melazolide B, a degraded limonoid, from a natural terpene precursor

Yannan Liu,† Alexander W. Schuppe,† Yizhou Zhao, Jaehoo Lee, Timothy R. Newhouse*

Department of Chemistry, Yale University, 225 Prospect St., New Haven, Connecticut 06520-8107, United States.
*E-mail: timothy.newhouse@yale.edu

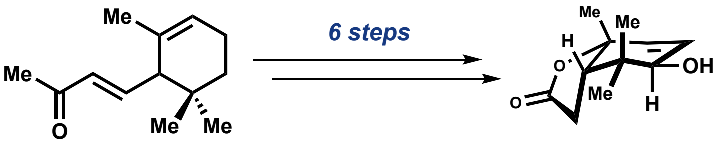

(±)-α-ionone (−)-melazolide B

Contents

General experimental SI2
Synthesis and spectroscopic data for triflate (**12**) SI3–SI5
Synthesis and spectroscopic data for alkene (**13**) SI6–SI8
Synthesis and spectroscopic data for C3-*epi*-melazolide B (**15**) SI12–SI14
Synthesis of enone (**17**) through a two-step sequence SI15
Synthesis and spectroscopic data for (–)-actinidiolide (**6**) SI16–SI20
Synthesis and spectroscopic data for (–)-melazolide B (**5**) SI21–SI24
References SI25

General Experimental

General Experimental Procedures: All reactions were carried out under an inert nitrogen atmosphere with dry solvents under anhydrous conditions unless otherwise stated. All reactions were capped with a rubber septum, or Teflon-coated silicon microwave cap unless otherwise stated. Stainless steel cannula or syringe were used to transfer solvent, and air- and moisture- sensitive liquid reagents. Reactions were monitored by thin-layer chromatography (TLC) and carried out on 0.25 mm Merck silica gel plates (60F-254) using UV light as the visualizing agent and potassium permanganate or an acidic solution of *p*-anisaldehyde as developing agents. Flash column chromatography employed SiliaFlash® P60 (40-60 μm, 230-400 mesh) silica gel purchased from SiliCycle Inc. Ozonolysis reactions were conducted using an A2Z Ozone 856689005177 SP - 16G Swimming Pool Ozone Generator with a 16000 MG/H adjustable ozone concentration.

Materials: All reaction solvents were purified using a Seca solvent purification system by Glass Contour. N,N-diisopropylamine, and triethylamine were distilled over CaH_2. *n*-BuLi (2.5 M in hexanes), $Zn(TMP)_2$ (0.5 M in toluene), and KHMDS were purchased from Sigma-Aldrich. $[Pd(allyl)Cl]_2$, and $[Rh(COD)(OH)]_2$, $Pd(OAc)_2$, and $Pd(TFA)_2$ were purchased from Strem Chemicals. (*S*,*S*)-(+)-N,N'-Bis(3,5-di-tert-butylsalicylidene)-1,2-cyclohexanediaminomanganese(III) chloride was purchased from Combi-Blocks. SeO_2 was purchased from Oakwood, and ground to a fine powder prior to use. Commercial concentrated Clorox® bleach solution was used as received. The molarity of *n*-butyllithium solutions were determined by titration with N-benzylbenzamide. All other reagents were used as received without further purification, unless otherwise stated.

Instrumentation: All new compounds were characterized by means of ^{1}H NMR, ^{13}C NMR, FT-IR (thin film), and HR-MS. Copies of the ^{1}H- and ^{13}C-NMR spectra can be found at the end of each experimental procedure. NMR spectra were recorded using a Varian 400 MHz NMR spectrometer, Varian 500 MHz NMR spectrometer, or a Varian 600 MHz NMR spectrometer. All ^{1}H-NMR data are reported in δ units, parts per million (ppm), and were calibrated relative to the signals for residual chloroform (7.26 ppm) in deuterochloroform ($CDCl_3$) or residual benzene (7.16 ppm) in deuterobenzene (C_6D_6). All ^{13}C-NMR data are reported in ppm relative to $CDCl_3$ (77.16 ppm) and were obtained with ^{1}H decoupling unless otherwise stated. The following abbreviations or combinations thereof were used to explain the multiplicities: s = singlet, d = doublet, t = triplet, q = quartet, br = broad, m = multiplet, and a = apparent. All IR spectra were taken on an FT-IR/Raman Thermo Nicolet 6700. High resolution mass spectra (HR-MS) were recorded on a Bruker microTOF mass spectrometer using ESI-TOF (electrospray ionization-time of flight). Optical rotation data was obtained using a Perkin-Elmer 341 and Autopol VI automatic polarimeter. HPLC data was obtained using Agilent 1260 HPLC.

Synthesis of triflate (12):

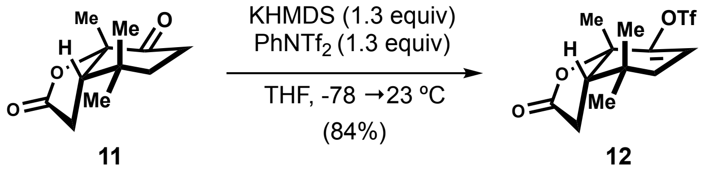

An evacuated flame-dried 25-mL round-bottomed flask equipped with a magnetic stir bar and a Schlenk adapter was brought into a glovebox. Once in the glovebox, the flask was filled with a nitrogen atmosphere. Solid KHMDS (330.0 mg, 1.66 mmol, 1.3 equiv) was added and the flask was sealed. The flask was removed from the glovebox, and the flask was evacuated and backfilled with nitrogen, this process was repeated three times. The Schlenk adapter was replaced with a rubber septum and the flask was placed under a nitrogen atmosphere. To this flask was added THF (2.5 mL, 0.66 M) via cannula, and the reaction mixture was allowed to stir at room temperature for 5 minutes to allow for complete dissolution of KHMDS solid. The reaction vessel was transferred to a –78 °C saturated dry-ice acetone bath and stirred for 10 minutes before a solution of ketone **11** (250.0 mg, 1.27 mmol, 1.0 equiv) in THF (2.5 mL, 0.5 M) was added dropwise over 5 minutes, resulting in a yellow reaction mixture.

After stirring at this temperature for 30 minutes, a solution of N-phenyl-bis(trifluoromethanesulfonimide) (593 mg, 1.66 mmol, 1.3 equiv) in THF (1.7 mL, 1.0 M) was added dropwise over five minutes. The reaction mixture was allowed to stir at this temperature for 15 minutes before the reaction vessel was removed from dry-ice acetone bath, and the reaction mixture was allowed to slowly warm up to room temperature over 1.5 hours. The reaction mixture was diluted with sat. aq. NH_4Cl (10 mL) and the layers were separated. The aqueous layer was extracted with EtOAc (3 x 5 mL). The combined organic extracts were washed with brine (10 mL), dried over anhydrous Na_2SO_4, filtered, and concentrated under reduced pressure by rotary evaporation to provide a crude yellow oil. Purification by flash column chromatography on silica gel (10% Et_2O/hexanes to 40% Et_2O/hexanes) afforded **12** (276 mg, 84%) as a colorless solid.

R_f: 0.13 (25% Et_2O/hexanes, $KMnO_4$)

^{1}H NMR (500 MHz, $CDCl_3$): δ 5.90 (dd, *J* = 5.4, 3.3 Hz, 1H), 2.65 (dd, *J* = 17.2, 8.3 Hz, 1H), 2.47–2.36 (m, 2H), 2.31 (dd, *J* = 18.4, 3.2 Hz, 1H), 2.10 (dd, *J* = 18.4, 5.4 Hz, 1H), 1.65 (s, 3H), 1.13 (s, 3H), 1.01 (s, 3H)

^{13}C NMR (126 MHz, $CDCl_3$): δ 173.7, 146.6, 119.7, 82.0, 52.5, 34.9, 32.2, 32.0, 28.3, 27.3, 24.2

IR (cm^{-1}): 1784, 1416, 1210, 1141, 1047, 949, 879, 612

ESI-HRMS (m/z): $[M+H]^+$ calc'd for $C_{12}H_{16}F_3O_5S^+$: 329.0665; found: 329.0659

$[\alpha]_D^{20.0}$: –6.32 ° (*c* 0.5, $CHCl_3$)

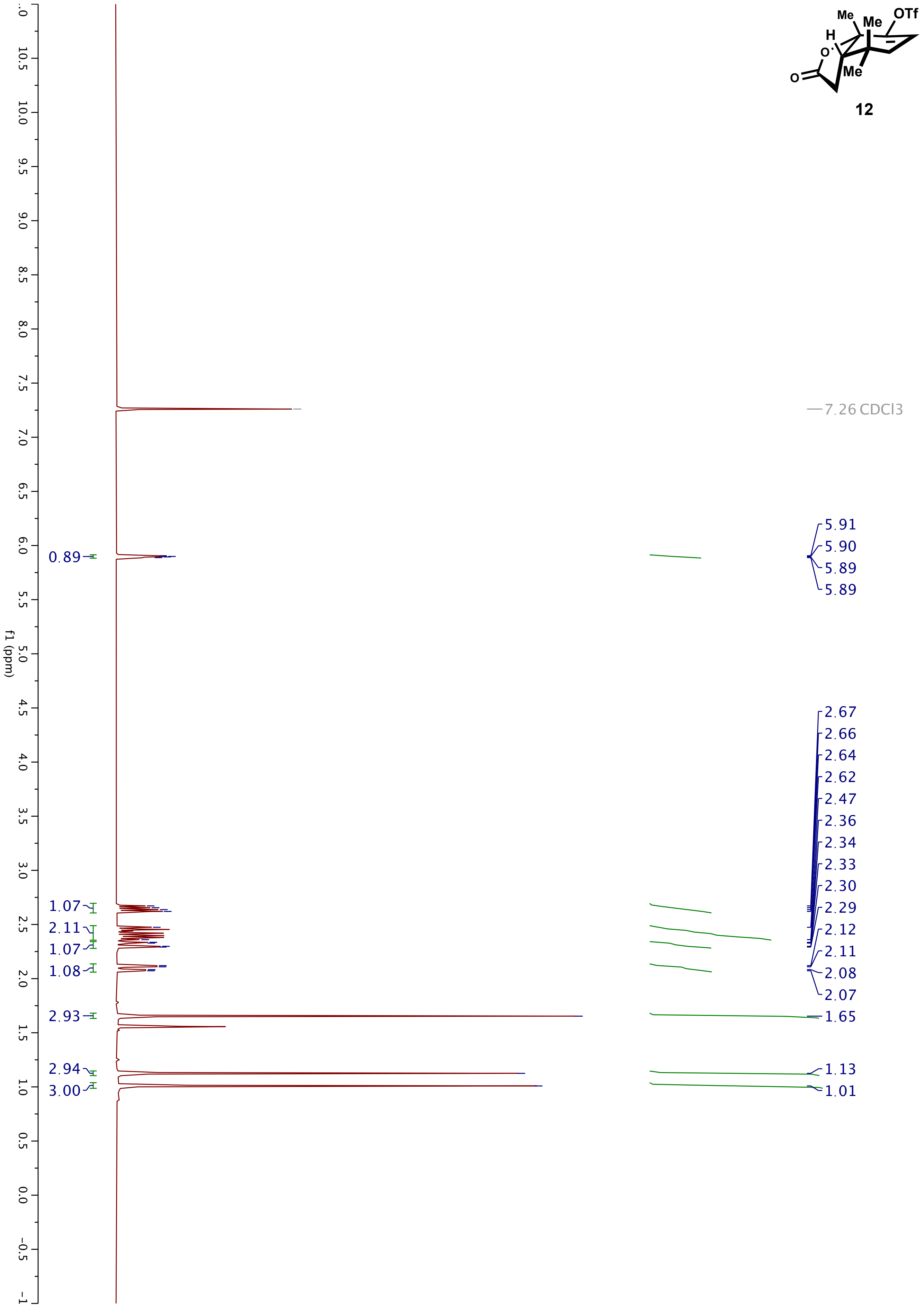

Me
Me
OTf
H
O
O
Me
12
7.26 CDCl3
5.91
5.90
5.89
5.89
2.67
2.66
2.64
2.62
2.47
2.36
2.34
2.33
2.30
2.29
2.12
2.11
2.08
2.07
1.65
1.13
1.01
0.89
1.07
2.11
1.07
1.08
2.93
2.94
3.00
f1 (ppm)

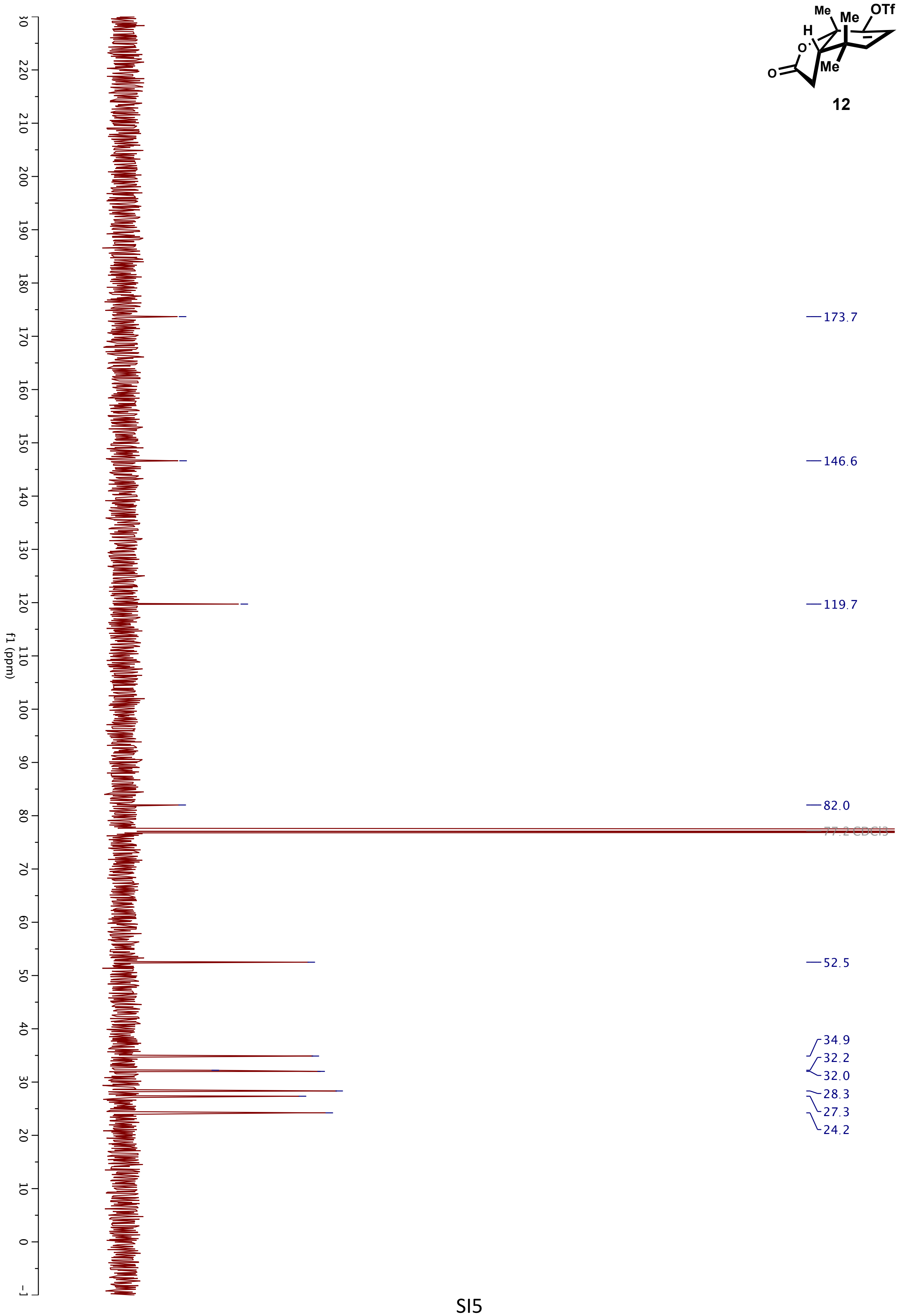
Me
Me
OTf
H
O
O
Me
12
173.7
146.6
119.7
82.0
77.2 CDCl3
52.5
34.9
32.2
32.0
28.3
27.3
24.2
f1 (ppm)

Synthesis of alkene (13):

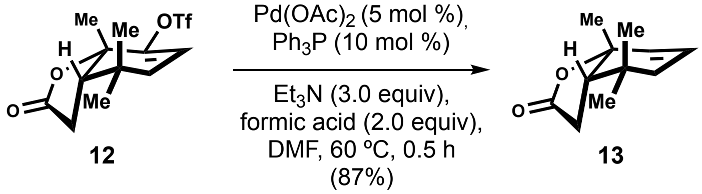

To a flame-dried 20-mL microwave vial equipped with a magnetic stir bar was added $Pd(OAc)_2$ (12.9 mg, 0.057 mmol, 5 mol %), and triphenylphosphine (30.2 mg, 0.115 mmol, 10 mol %). The reaction vial was sealed with a Teflon-coated silicon microwave cap, and the reaction vial was evacuated and backfilled with nitrogen, this process was repeated three times. A degassed solution of triflate **12** (280 mg, 1.15 mmol, 1.0 equiv) in DMF (2.3 mL, 0.5 M, degassed by freeze-pump-thaw 3 x 10 minutes) was added to the reaction mixture at room temperature. To the reaction mixture was added Et_3N (0.48 mL, 3.4 mmol, 3.0 equiv) and formic acid (87 µL, 2.3 mmol, 2.0 equiv). The reaction vessel was moved to a 60 °C preheated oil bath.

After the reaction mixture had stirred at 60 °C for 30 min, the reaction vessel was removed from the oil bath and cooled to room temperature. Once at room temperature, the reaction mixture was diluted with water (10 mL) and EtOAc (5 mL). The layers were separated and the aqueous phase was extracted with EtOAc (2 x 5 mL). The combined organic extracts were washed with brine (2 x 10 mL), dried with Na_2SO_4, filtered, and concentrated under reduced pressure by rotary evaporation. Purification by flash column chromatography on silica gel (15% EtOAc/hexanes) afforded alkene **13** (156 mg, 87%) as a colorless oil.

R_f: 0.1 (15% Et_2O/hexanes, *p*-anisaldehyde)

^{1}H NMR (500 MHz, $CDCl_3$): δ 5.78 (ddd, *J* = 10.2, 4.8, 2.9 Hz, 1H), 5.67 (d, *J* = 10.2 Hz, 1H), 2.55 (dd, *J* = 17.6, 8.8 Hz, 1H), 2.42 (dd, *J* = 17.6, 10.1 Hz, 1H), 2.19 (t, *J* = 9.4 Hz, 1H), 2.03 (dt, *J* = 18.2, 2.8 Hz, 1H), 1.81 (ddd, *J* = 18.2, 4.9, 1.6 Hz, 1H), 1.53 (s, 3H), 1.03 (s, 3H), 0.95 (s, 3H)

^{13}C NMR (126 MHz, $CDCl_3$): δ 175.8, 128.5, 128.1, 84.4, 50.5, 34.7, 32.7, 31.9, 28.6, 28.0, 27.5

IR (cm^{-1}): 1767, 1377, 1286, 1267, 1081, 940, 814, 741, 615

ESI-HRMS (m/z): $[M+H]^+$ calc'd for $C_{11}H_{17}O_2^+$: 181.1223; found: 181.1227

$[\alpha]_D^{20.0}$: –60.6 ° (*c* 1.0, $CHCl_3$)

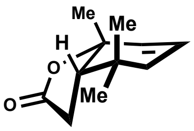

13

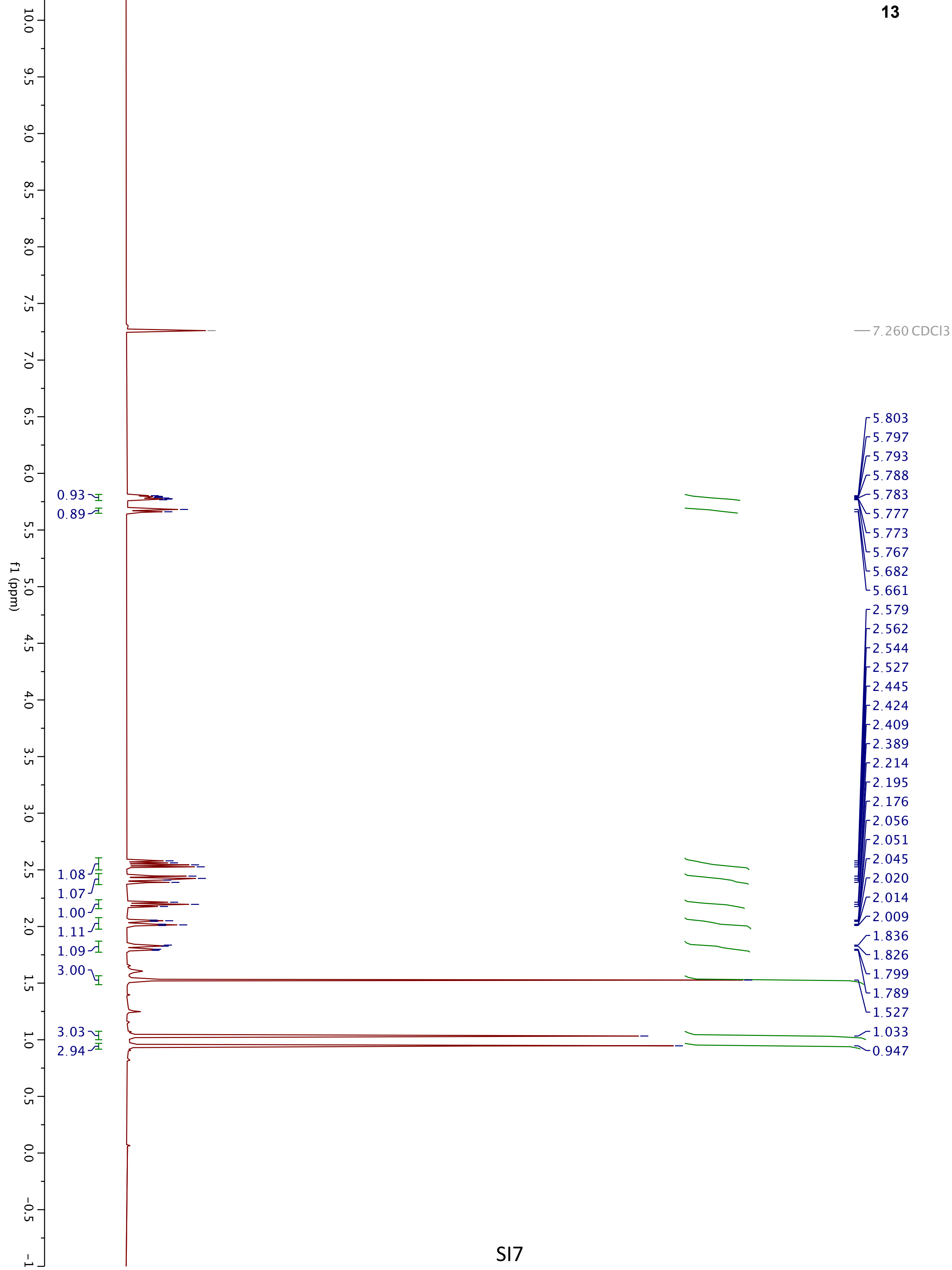

f1 (ppm)
7.260 CDCl3
5.803
5.797
5.793
5.788
5.783
5.777
5.773
5.767
5.682
5.661
2.579
2.562
2.544
2.527
2.445
2.424
2.409
2.389
2.214
2.195
2.176
2.056
2.051
2.045
2.020
2.014
2.009
1.836
1.826
1.799
1.789
1.527
1.033
0.947
0.93
0.89
1.08
1.07
1.00
1.11
1.09
3.00
3.03
2.94

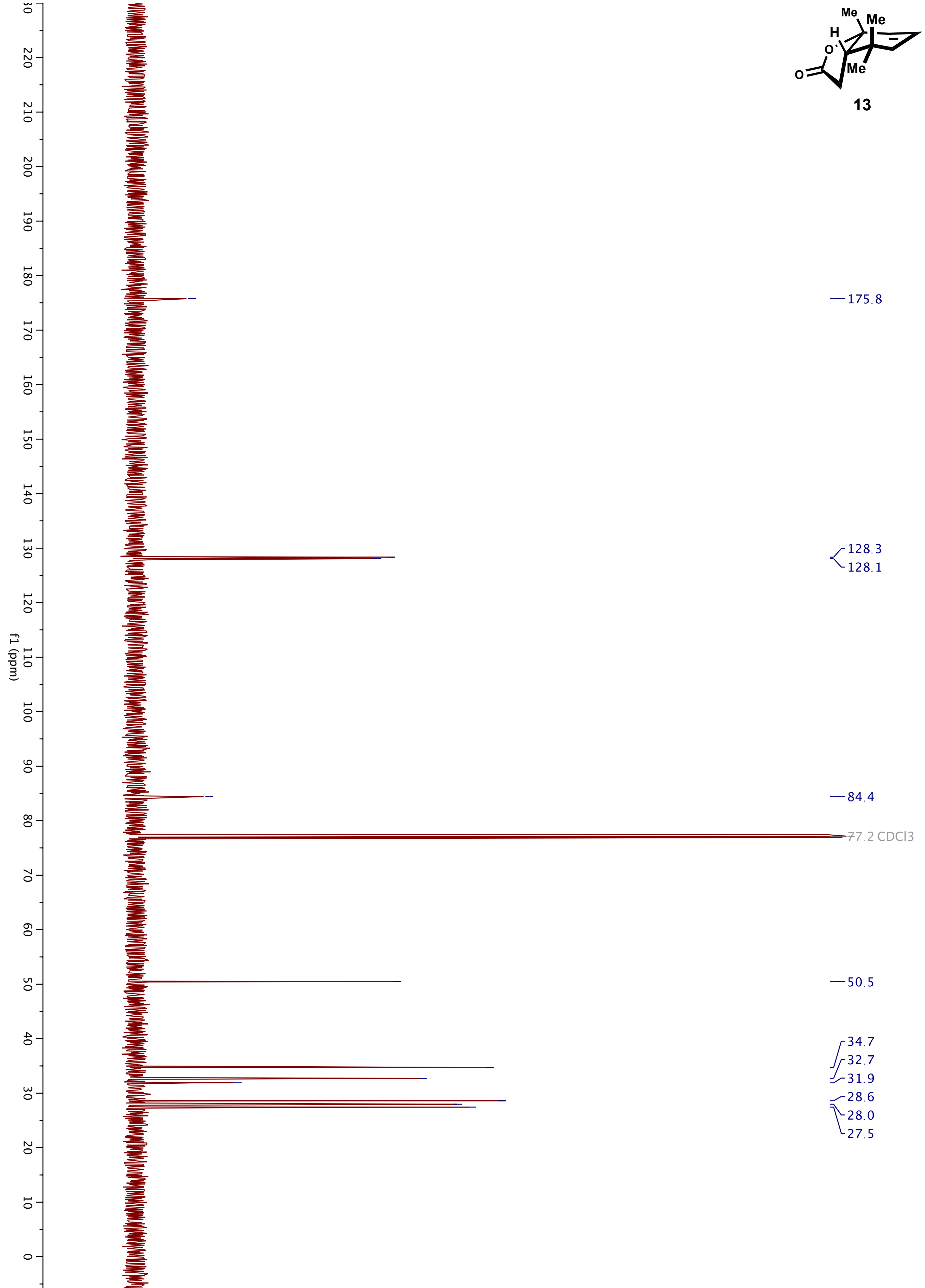
Me
Me
H
O
Me
O
13
175.8
128.3
128.1
84.4
77.2 CDCl3
50.5
34.7
32.7
31.9
28.6
28.0
27.5
f1 (ppm)

Synthesis of enone (14)

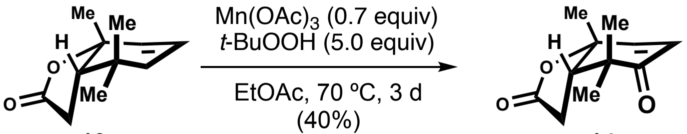

13 **14**

To a flame-dried 100 mL round-bottomed flask equipped with a magnetic stir bar was added $Mn(OAc)_3$ (780 mg, 2.9 mmol, 0.7 equiv) and lactone **13** (750 mg, 4.2 mmol, 1.0 eqiuv). The reaction flask was evacuated and backfilled with nitrogen, and this process was repeated for a total of three times. To the reaction vessel was added EtOAc (30 mL, 0.14 M) and TBHP (7.6 mL, 20.8 mmol, 5.0 equiv) at room temperature. The reaction vessel was fitted with an oven-dried reflux condenser, and the reaction apparatus was moved to an oil bath, which was heated to 70 °C.

After the reaction mixture had stirred at 70 °C for 3 days, the reaction apparatus was cooled to room temperature. Following this, the reaction mixture was diluted with sat. aq. $NaHCO_3$ (80 mL) and EtOAc (20 mL), and the layers were separated. The aqueous layer was extracted with EtOAc (3 x 30 mL). The combined organic extracts were washed with brine (100 mL), dried over anhydrous Na_2SO_4, filtered, and concentrated under reduced pressure by rotary evaporation to provide a crude yellow oil. Purification by flash column chromatography on silica gel (hexanes/EtOAc 2:1) afforded **14** (322 mg, 40%) as colorless oil.

R_f: 0.53 (hexanes/EtOAc 1:1, $KMnO_4$)

^{1}H NMR (500 MHz, $CDCl_3$): δ 6.56 (d, J = 10.0 Hz, 1H), 6.02 (d, J = 10.0 Hz, 1H), 2.70 (dd, J = 16.5, 8.5 Hz, 1H), 2.65 (dd, J = 18.5, 8.5 Hz, 1H), 2.28 (dd, J = 16.5, 11.0 Hz, 1H), 1.73 (s, 3H), 1.29 (s, 3H), 1.15 (s, 3H)

^{13}C NMR (126 MHz, $CDCl_3$): δ 201.4, 173.5, 144.3, 127.8, 82.3, 52.4, 44.4, 34.5, 27.4, 26.5, 23.4.

IR (cm^{-1}): 2980, 1787, 1683, 1378, 1244, 1142, 1084, 955, 669.

ESI-HRMS (m/z): $[M+H]^+$ calc'd for $C_{11}H_{15}O_3^+$: 195.1016; found: 195.1024

$[\alpha]_D^{20.0}$: –199.8 ° (c 0.15, $CHCl_3$).

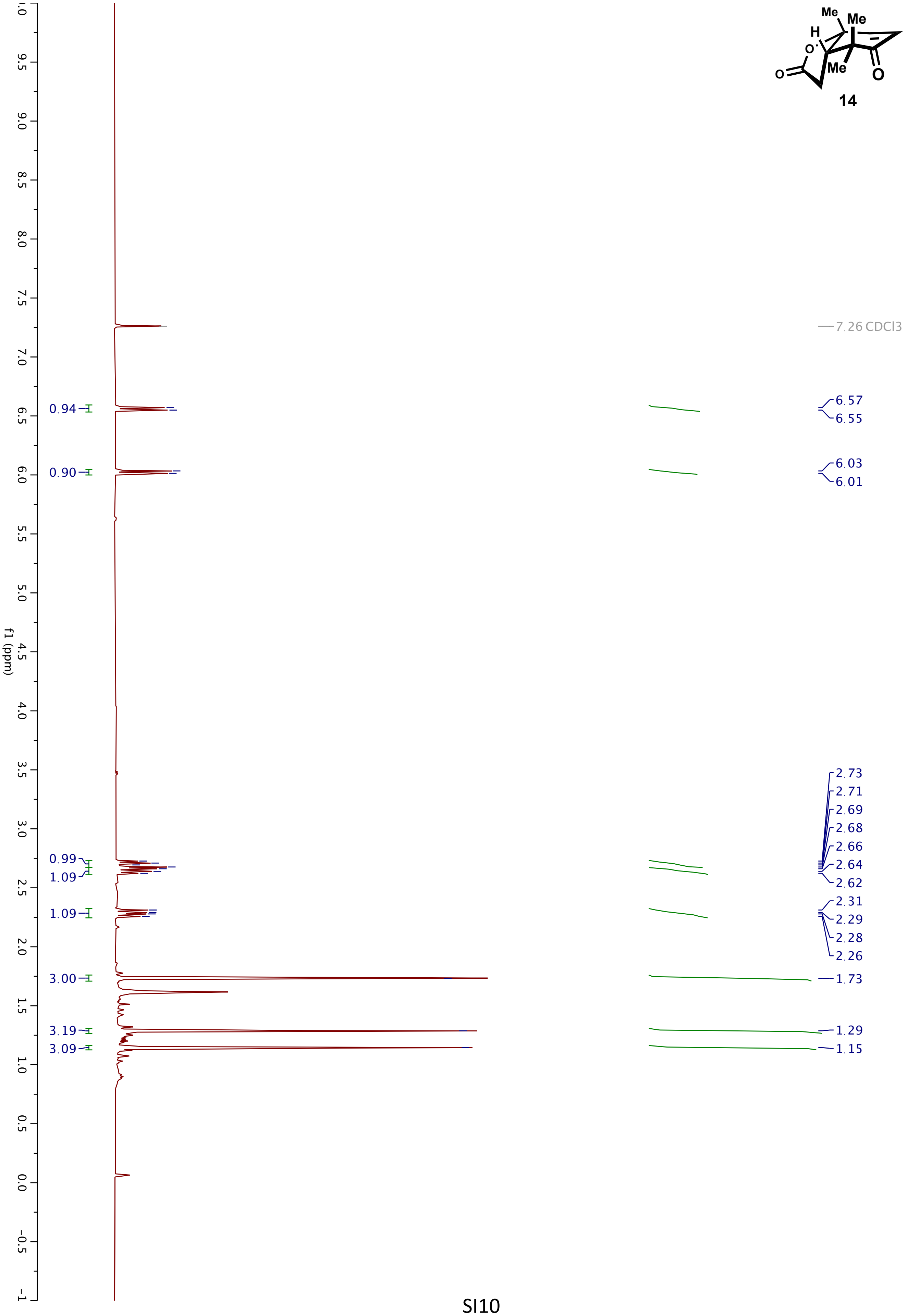
Me
Me
H
O
Me
O
O
14
7.26 CDCl3
6.57
6.55
6.03
6.01
2.73
2.71
2.69
2.68
2.66
2.64
2.62
2.31
2.29
2.28
2.26
1.73
1.29
1.15
0.94
0.90
0.99
1.09
1.09
3.00
3.19
3.09
f1 (ppm)

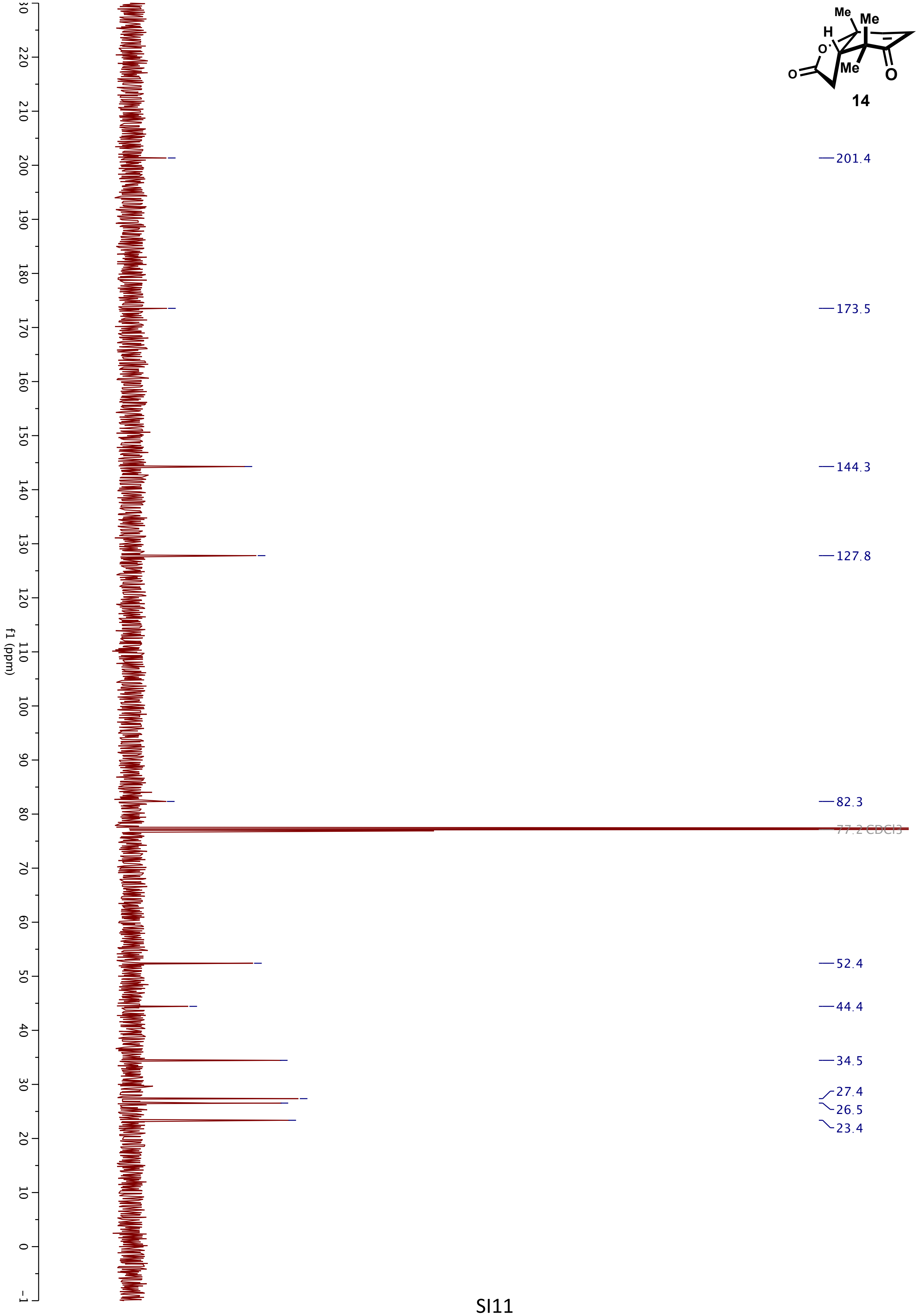

Me
Me
H
O
Me
O
O
14
201.4
173.5
144.3
127.8
82.3
77.2 CDCl3
52.4
44.4
34.5
27.4
26.5
23.4
f1 (ppm)

Synthesis of C3-*epi*-melazolide B (15):

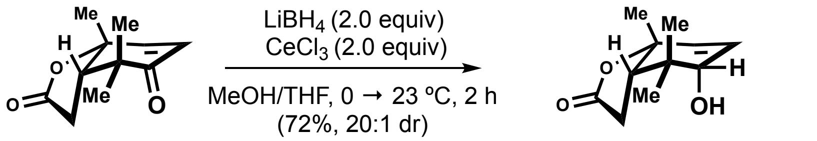

14 C3-*epi*-melazolide B (15)

To a flame-dried 10 mL microwave vial equipped with a magnetic stir bar was added enone **14** (38.8 mg, 0.2 mmol, 1.0 equiv) and $CeCl_3{\cdot}7H_2O$ (149.0 mg, 0.4 mmol, 2.0 equiv). The reaction vial was evacuated and backfilled with nitrogen, and this process was repeated for a total of three times. To this flask was added THF/MeOH (2 mL, 1:1, 0.1 M), and the reaction vessel was transferred to a 0 °C ice-water bath.

After the reaction mixture had stirred for 10 minutes at 0 °C, $LiBH_4$ (0.2 mL, 0.4 mmol, 2.0 equiv, 2.0 M in THF) was added, which resulted in a yellow solution. After stirring for an additional 30 minutes at 0 °C, the reaction vessel was allowed to warm to room temperature. After stirring at room temperature for 1 hour, the reaction mixture was diluted with sat. aq. NH_4Cl (15 mL) and EtOAc (5 mL), and the layers were separated. The aqueous layer was extracted with EtOAc (3 x 5 mL). The combined organic extracts were washed with brine (20 mL), dried over anhydrous Na_2SO_4, filtered, and concentrated under reduced pressure by rotary evaporation to provide a crude yellow foam. Purification by flash column chromatography on silica gel (1:1.5 hexanes/EtOAc) afforded **15** (28.3 mg, 72%, 20:1 dr) as a colorless oil.

R_f: 0.40 (hexanes/EtOAc 1:3, $KMnO_4$)

^{1}H NMR (400 MHz, $CDCl_3$): δ 5.54–5.47 (m, 2H), 4.45 (s, 1H), 2.57 (dd, J = 16.0, 7.6 Hz, 1H), 2.47 (d, J = 12.4 Hz, 1H), 2.42–2.36 (m, 1H), 1.46 (s, 3H), 1.15 (s, 3H), 1.02 (s, 3H)

^{13}C NMR (126 MHz, $CDCl_3$): δ 175.7, 137.2, 125.8, 88.9, 71.1, 52.1, 35.0, 33.8, 31.3, 27.2, 21.1.

IR (cm^{-1}): 3447, 2925, 1776, 1669, 1465, 1379, 1260, 1172, 1057, 944

ESI-HRMS (m/z): $[M+H]^+$ calc'd for $C_{11}H_{17}O_3^+$: 197.1172; found: 197.1174

$[\alpha]_D^{20.0}$: –199.8 ° (c 0.15, $CHCl_3$). $[\alpha]_D^{20.0}$: –32.2 ° (c 0.025, $CHCl_3$).

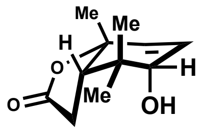

C3-*epi*-melazolide B (15)

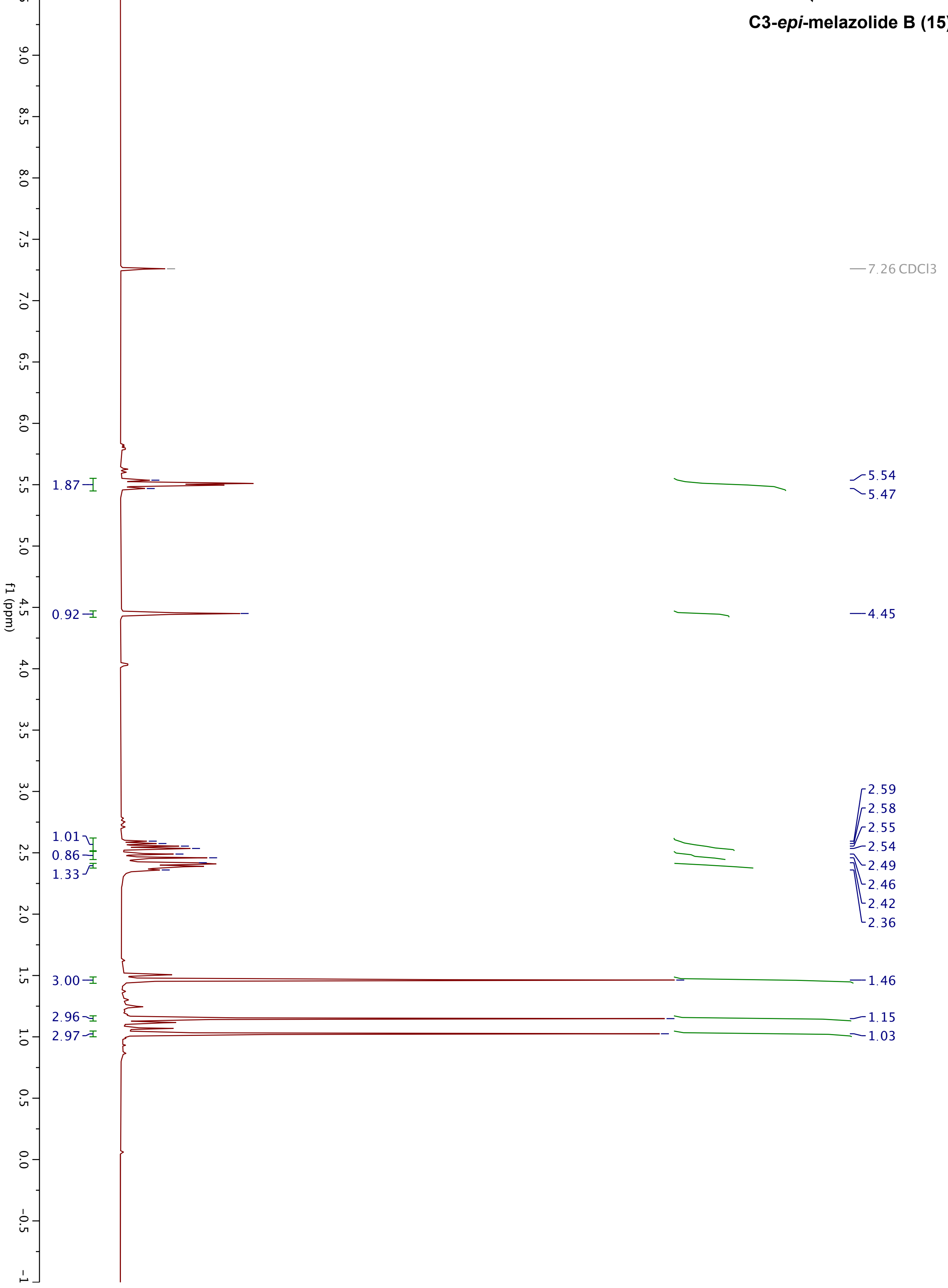

C3-*epi*-melazolide B (15)

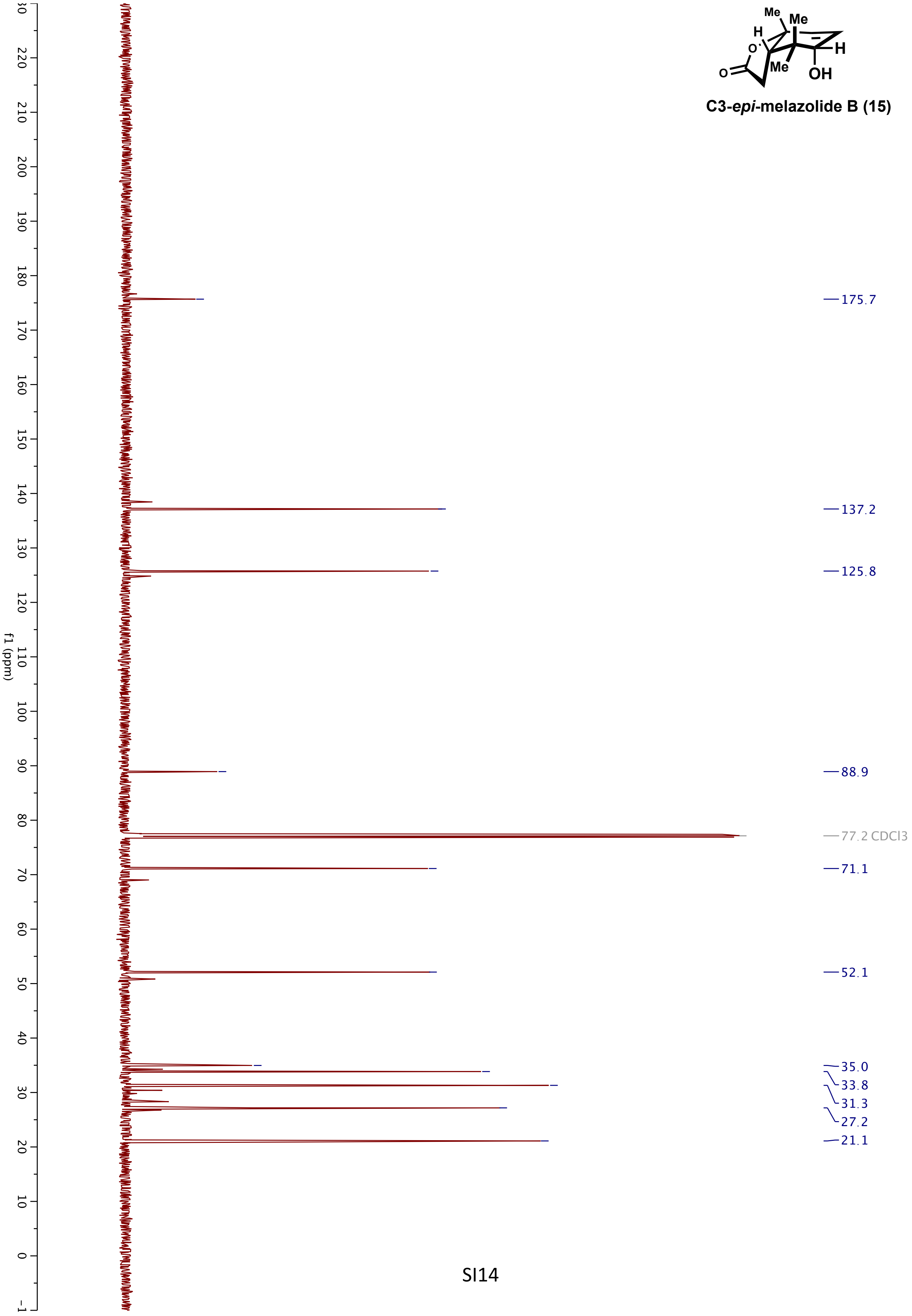

Synthesis of enone (17) through a two-step sequence:

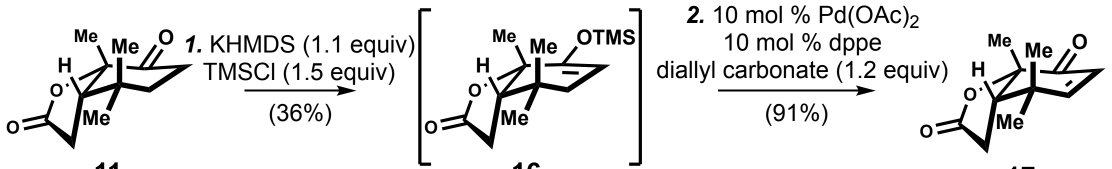

11 16 17

An evacuated flame-dried 250-mL round-bottomed flask equipped with a magnetic stir bar and a Schlenk adapter was brought into a glovebox. Once in the glovebox, the flask was filled with a nitrogen atmosphere. Solid KHMDS (3.4 g, 17.1 mmol, 1.1 equiv) was added and the flask was sealed. The flask was removed from the glovebox, and the flask was evacuated and backfilled with nitrogen three times. The Schlenk adapter was replaced with a rubber septum and placed under a nitrogen atmosphere. To this flask was added THF (105 mL, 0.15 M) via cannula, and this mixture was allowed to stir at room temperature for 5 minutes to allow for complete dissolution of KHMDS solid. The reaction vessel was transferred to a –78 °C saturated dry-ice acetone bath and stirred for 10 minutes before a solution of ketone **11** (3.1 mg, 15.6 mmol, 1.0 equiv) in THF (31 mL, 0.5 M) was added over 10 minutes, resulting in a yellow reaction mixture. After stirring at this temperature for 30 minutes, chlorotrimethylsilane (3.0 mL, 23.4 mmol, 1.5 equiv) was added dropwise over 5 minutes. The reaction mixture was allowed to stir at this temperature for 30 minutes before the reaction vessel was removed from dry-ice acetone bath, and the reaction mixture was allowed to slowly warm up to room temperature over 1.5 hours. The reaction mixture was diluted with sat. aq. NH_4Cl (100 mL) and the layers were separated. The aqueous layer was extracted with EtOAc (3 x 100 mL). The combined organic extracts were washed with brine (100 mL), dried over anhydrous Na_2SO_4, filtered, and concentrated under reduced pressure by rotary evaporation to provide a crude yellow oil. Purification by flash column chromatography on silica gel (10% Et_2O/hexanes to 30% Et_2O/hexanes) afforded **16** (1.5 g, 36%) as a yellow oil.

To a flame-dried 100-mL Schlenk flask equipped with a magnetic stir bar was added $Pd(OAc)_2$ (75 mg, 0.335 mmol, 10 mol %), and 1,2-bis(diphenylphosphino)ethane (133 mg, 0.335 mmol, 10 mol %). The flask was evacuated and backfilled with nitrogen, and this process was repeated for a total of three times. To the reaction mixture was added a solution of silyl enol ether **16** (900. mg, 3.35 mmol, 1.0 equiv) in acetonitrile (33.5 mL, 0.1 M), and diallyl carbonate (0.58 mL, 4.02 mmol, 1.2 equiv). The reaction vessel was moved to an 85 °C preheated oil bath. After the reaction mixture had stirred at 85 °C for 4 hours, the reaction vessel was removed from the oil bath and cooled to room temperature. Once at room temperature, the reaction mixture was diluted with sat. aq. NH_4Cl (30 mL) and EtOAc (30 mL). The layers were separated and the aqueous phase was extracted with EtOAc (2 x 30 mL). The combined organic extracts were washed with brine (30 mL), dried with Na_2SO_4, filtered, and concentrated under reduced pressure by rotary evaporation. Purification by flash column chromatography on silica gel (EtOAc/hexanes 1:2) afforded enone **17** (591 mg, 91%) as a colorless oil. The spectral data matched that which was previously reported.[1]

Synthesis of (–)-actinidiolide (6):

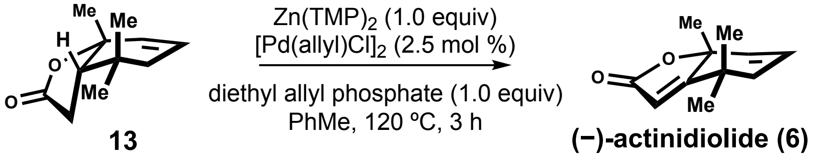

To a flame-dried 10-mL microwave vial equipped with a magnetic stir bar, was added lactone **15** (36.0 mg, 0.20 mmol, 1.0 equiv) and toluene (1.0 mL, 0.20 M). The reaction mixture was cooled to 0 °C by transferring the reaction apparatus to an ice-water bath. To the above solution was added $Zn(TMP)_2$ (0.40 mL, 0.50 M in toluene, 0.20 mmol, 1.0 equiv). The reaction mixture was stirred for 10 min at the same temperature. A freshly prepared solution of $[Pd(allyl)Cl]_2$ (1.8 mg, 0.0050 mmol, 0.025 equiv) and diethyl allyl phosphate (36.0 µL, 0.20 mmol, 1.0 equiv) in toluene (0.2 mL) was next added at 0 °C. The reaction mixture was removed from the ice bath and put into a 120 °C preheated oil bath.

After the reaction mixture had stirred at 120 °C for 3 hours, the resulting reaction vessel was removed from the oil bath and cooled to room temperature. To the reaction mixture was added sat. aq. NH_4Cl (6 mL), diluted with EtOAc (5 mL), and the organic phase was separated. The aqueous phase was extracted with EtOAc (3 × 5 mL) and the combined organic layers were washed with brine (15 mL), dried over anhydrous Na_2SO_4, filtered, and concentrated under reduced pressure by rotary evaporation. Purification by flash column chromatography on silica gel (hexane/EtOAc = 7:1) afforded **6** (30.2 mg, 85%) as a colorless solid.

R_f: 0.13 (15% Et_2O/hexanes, *p*-anisaldehyde)

^{1}H NMR (400 MHz, $CDCl_3$): 5.89 (dd, *J* = 9.8, 2.1 Hz, 1H), 5.74 (ddd, *J* = 9.8, 4.7, 2.8 Hz, 1H), 5.71 (s, 1H), 2.27 (ddd, *J* = 17.7, 4.6, 1.4 Hz, 1H), 2.14 (ddd, *J* = 17.7, 2.8, 2.8 Hz, 1H), 1.60 (s, 3H), 1.33 (s, 3H), 1.29 (s, 3H)

^{13}C NMR (101 MHz, $CDCl_3$): δ 180.9, 171.5, 128.9, 128.4, 112.7, 85.5, 44.5, 35.8, 28.2, 26.2, 26.1

IR (cm^{-1}): 1750, 1630, 1256, 1081, 953, 858, 742

ESI-HRMS (m/z): $[M+H]^+$ calc'd for $C_{11}H_{15}O_2^+$: 179.1067; found: 179.1068

$[\alpha]_D^{20.0}$**:** –129.8 ° (*c* 1.0, $CHCl_3$) Lit: –149.9 ° (*c* 0.8, $CHCl_3$)

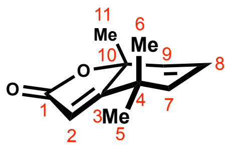

(–)-actinidiolide (6)

Table SI-1: Spectroscopic Comparison of (–)-actinidiolide (**6**) by ^{1}H-NMR

**Carbon #**	**This Report: actinidiolide (CDCl$_3$, 400 MHz)**	**Jørgensen & Co-workers: actinidiolide (CDCl$_3$, 90 MHz)[2]**	**Difference**
**1**			
**2**	5.71 (s)	5.72 (s)	0.01
**3**			
**4**			
**5**	1.29 (s)	1.31 (s)	0.02
**6**	1.33 (s)	1.34 (s)	0.01
**7 (α)**	2.14 (ddd, *J* = 17.7, 2.8, 2.8 Hz	2.16 (ddd, *J* = 17.8, 2.7, 2.4 Hz)	0.02
**7 (β)**	2.27 (ddd, *J* = 17.7, 4.6, 1.4 Hz)	2.28 (ddd, *J* = 17.8, 4.5, 1.2 Hz)	0.01
**8**	5.74 (ddd, *J* = 9.8,4.7, 2.8 Hz)	5.76 (ddd, *J* = 10.0, 4.5, 2.7 Hz)	0.02
**9**	5.89 (dd, *J* = 9.8, 2.1 Hz)	5.90 (d, *J* = 10.0 Hz)	0.01
**10**			
**11**	1.60 (s)	1.62 (s)	0.02

The ^{1}H-NMR data for synthetic actinidiolide listed in the table above are reported in ppm relative to $CDCl_3$ calibrated to 7.26 ppm.

(–)-Actinidiolide (**6**)
Jørgensen & Co-workers
(*J. Org. Chem.* **1998**, *63*, 118 – 121): **(–)-actinidiolide (6)**

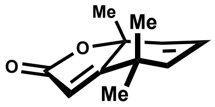

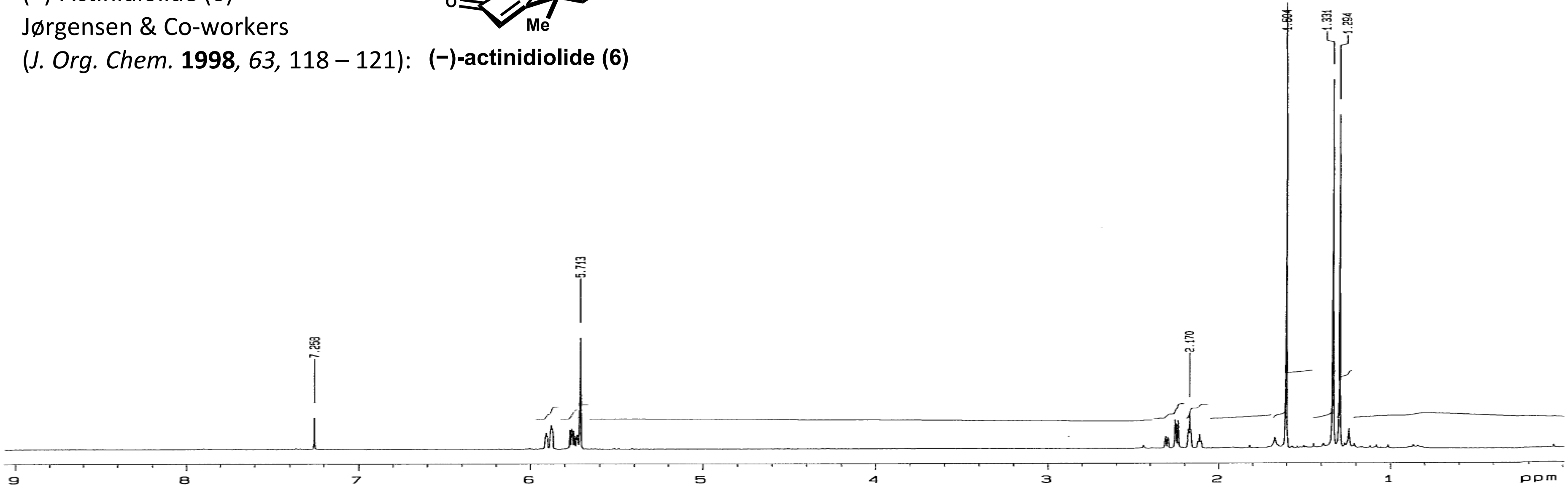

(–)-Actinidiolide (**6**)
400 MHz, this work:

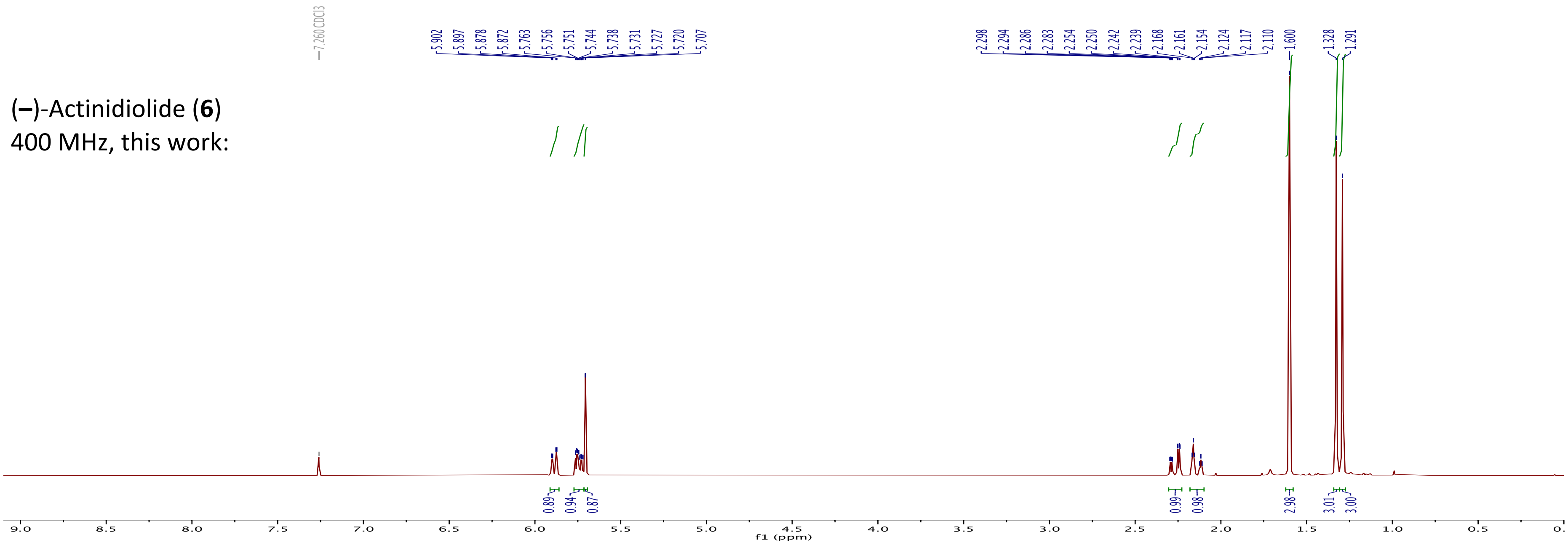

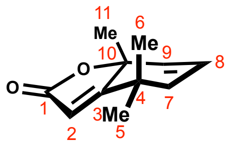

(–)-actinidiolide (6)

Table SI-2: Spectroscopic Comparison of (–)-actinidiolide (**6**) by ^{13}C-NMR

Carbon #	This Report: actinidiolide ($CDCl_3$, 400 MHz)	Jørgensen & Co-workers: actinidiolide ($CDCl_3$, 90 MHz)[2]	Difference
**1**	171.5	171.5	0.0
**2**	112.7	112.7	0.0
**3**	180.9	180.9	0.0
**4**	35.8	35.8	0.0
**5**	26.1	26.1	0.0
**6**	26.2	26.2	0.0
**7**	44.5	44.5	0.0
**8**	128.4	128.5	0.1
**9**	128.9	128.9	0.0
**10**	85.5	85.5	0.0
**11**	28.2	28.3	0.1

The ^{13}C-NMR data for synthetic actinidiolide listed in the table above are reported in ppm relative to $CDCl_3$ calibrated to 77.03 ppm.

Natural (–)-actinidiolide (**6**)
Jørgensen & Co-workers
(*J. Org. Chem.* **1998**, *63*, 118 – 121): **(−)-actinidiolide (6)**

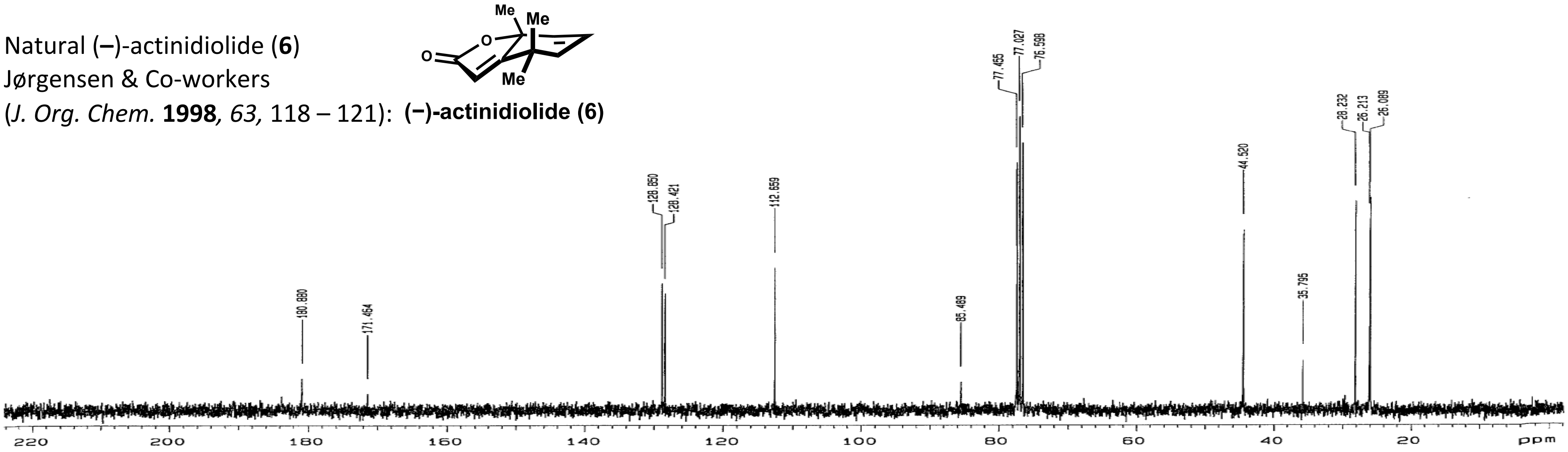

Synthetic (–)-actinidiolide (**6**)
101 MHz, this work:

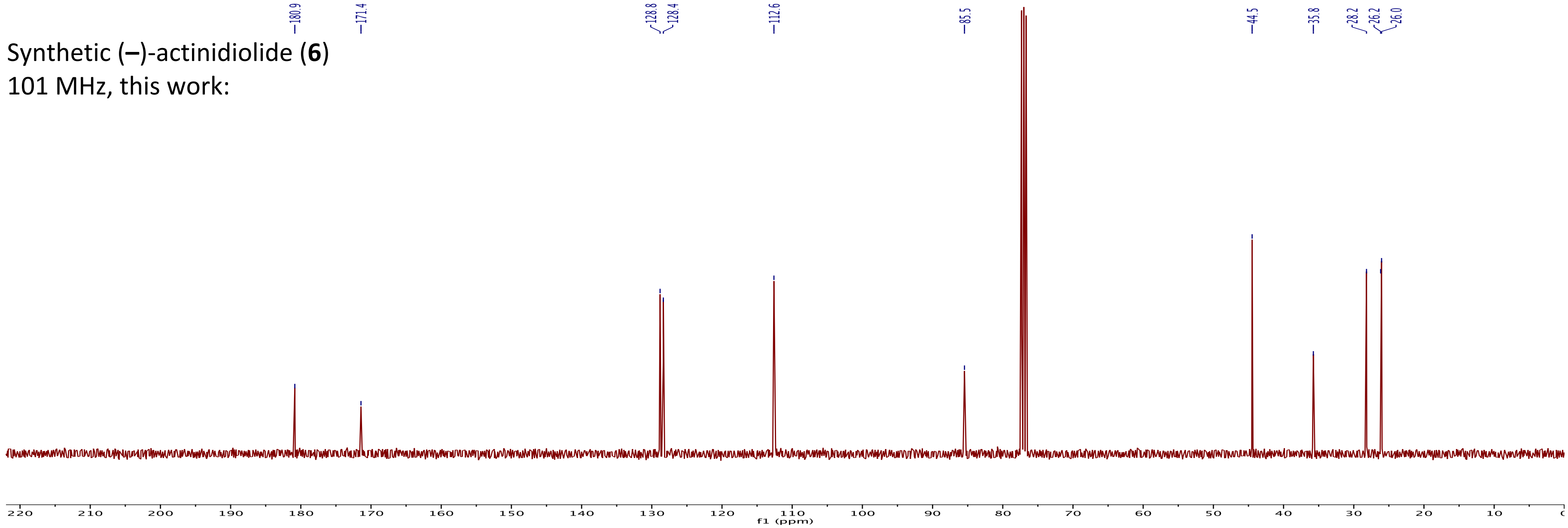

Synthesis of (–)-melazolide B (5)

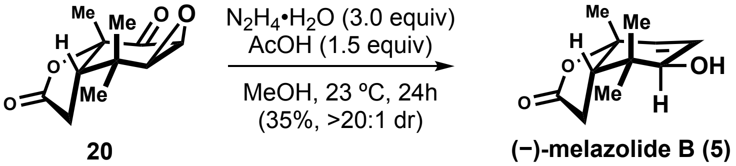

To a flame-dried 10 mL microwave vial equipped with a magnetic stir bar was added epoxide **22** (42.0 mg, 0.2 mmol, 1.0 equiv) and $N_2H_4 \cdot H_2O$ (29.1 mg, 0.6 mmol, 3.0 equiv). The reaction vial was evacuated and backfilled with nitrogen, and this process was repeated for a total of three times. To this flask was added MeOH (2 mL, 0.1 M) and AcOH (17.2 mL, 0.3 mmol, 1.5 equiv) at room temperature.

After stirring at room temperature for 24 hours, the reaction mixture was diluted with sat. aq. NH_4Cl (15 mL) and EtOAc (5 mL), and the layers were separated. The aqueous layer was extracted with EtOAc (3 x 5 mL). The combined organic extracts were washed with brine (20 mL), dried over anhydrous Na_2SO_4, filtered, and concentrated under reduced pressure by rotary evaporation to provide a crude yellow foam. Purification by flash column chromatography on silica gel (1:2 hexanes/EtOAc) afforded **5** (13.7 mg, 35%, >20:1 dr) as a colorless oil.

R$_f$: 0.41 (hexanes/EtOAc 1:3, $KMNO_4$)

^{1}H NMR (500 MHz, $CDCl_3$): δ 5.86 (dd, *J* = 10.0, 3.0 Hz, 1H), 5.77 (dd, *J* = 10.0, 1.5 Hz, 1H), 4.05 (dd, *J* = 3.0, 1.5 Hz, 1H), 2.66 (dd, *J* = 17.0, 8.0 Hz, 1H), 2.50–2.41 (m, 2H), 1.54 (s. 3H), 1.03 (s. 3H), 1.01 (s. 3H)

^{13}C NMR (126 MHz, CDCl3): δ 175.1, 131.3, 130.0, 83.5, 71.0, 49.0, 36.7, 31.6, 27.5, 24.3, 22.7

IR (cm^{-1}): 3448, 2928, 1755, 1377, 1290, 1238, 1080, 942, 815, 757, 669

ESI-HRMS (m/z): $[M+H]^+$ calc'd for $C_{11}H_{16}O_3^+$: 197.1172; found: 197.1182

$[\alpha]_D^{20.0}$**:** –144.4 ° (*c* 0.1, $CHCl_3$) –297 ° (*c* 0.027, EtOH) Lit: –75 ° (*c* 0.027, EtOH)

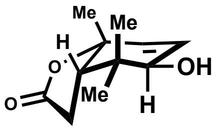

(−)-melazolide B (5)

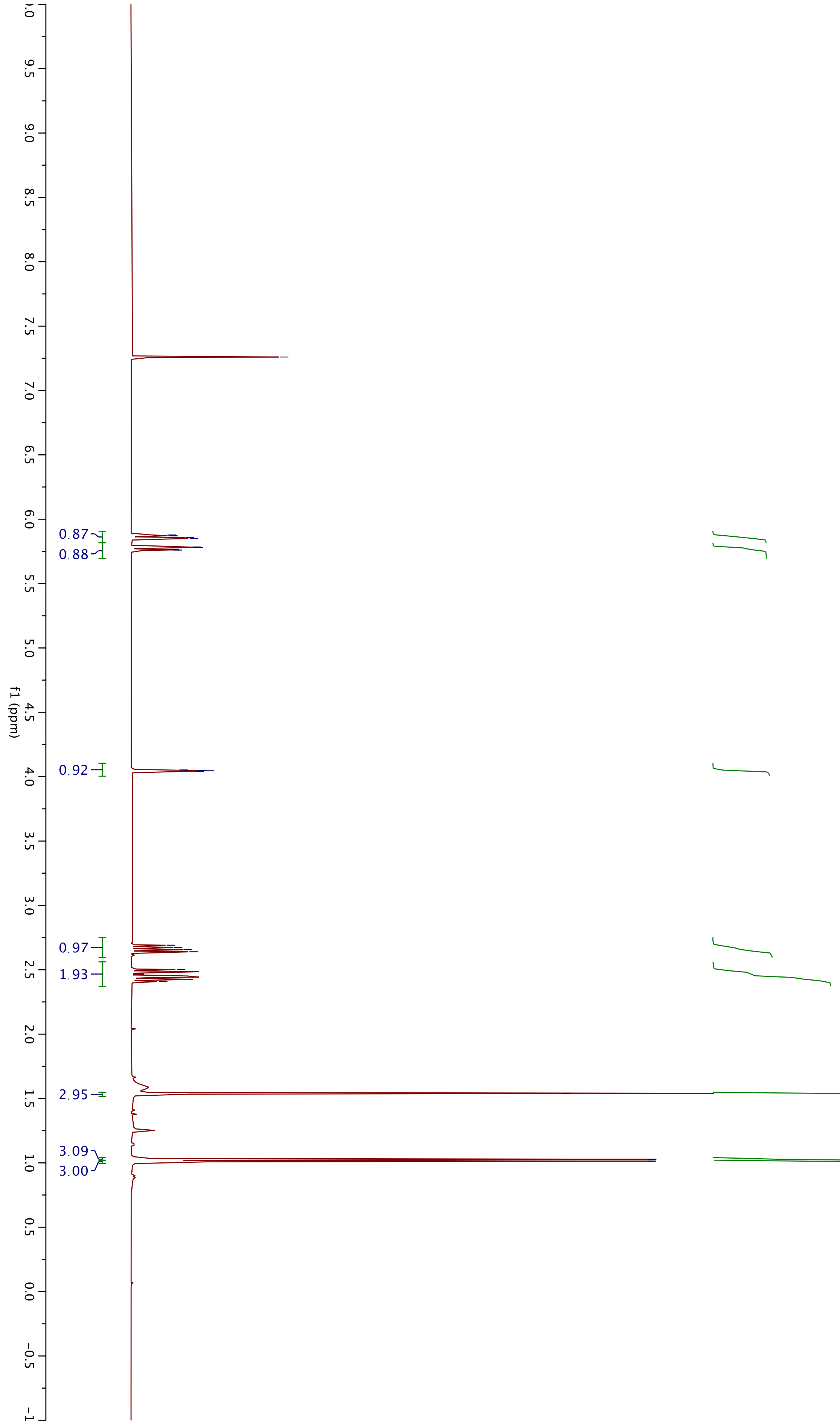

**This Report:**<br>**(–)-melazolide B**<br>**($CDCl_3$, 500 MHz)**	**D'Ambrosio & Co-workers:**<br>**(–)-melazolide B**<br>**-**	**Absolute**<br>**Difference**
5.86 (dd, *J* = 10.0, 3.0 Hz, 1H)	5.85 (dd, *J* = 10.0, 3.2 Hz, 1H)	0.01
5.77 (dd, *J* = 10.0, 1.5 Hz, 1H)	5.77 (dd, *J* = 10.0, 1.2 Hz, 1H)	0.00
4.05 (dd, *J* = 3.0, 1.5 Hz, 1H)	4.04 (dd, *J* = 3.2, 1.2 Hz, 1H)	0.00
2.66 (dd, *J* = 17.0, 8.0 Hz, 1H)	2.65 (m, 1H)	0.01
2.50–2.41 (m, 2H)	2.45 (m, 2H)	-
1.54 (s. 3H)	1.53 (s. 3H)	0.01
1.03 (s. 3H)	1.02 (s. 3H)	0.01
1.01 (s. 3H)	1.00 (s. 3H)	0.01

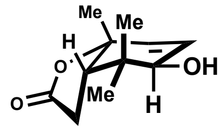

(−)-melazolide B (5)

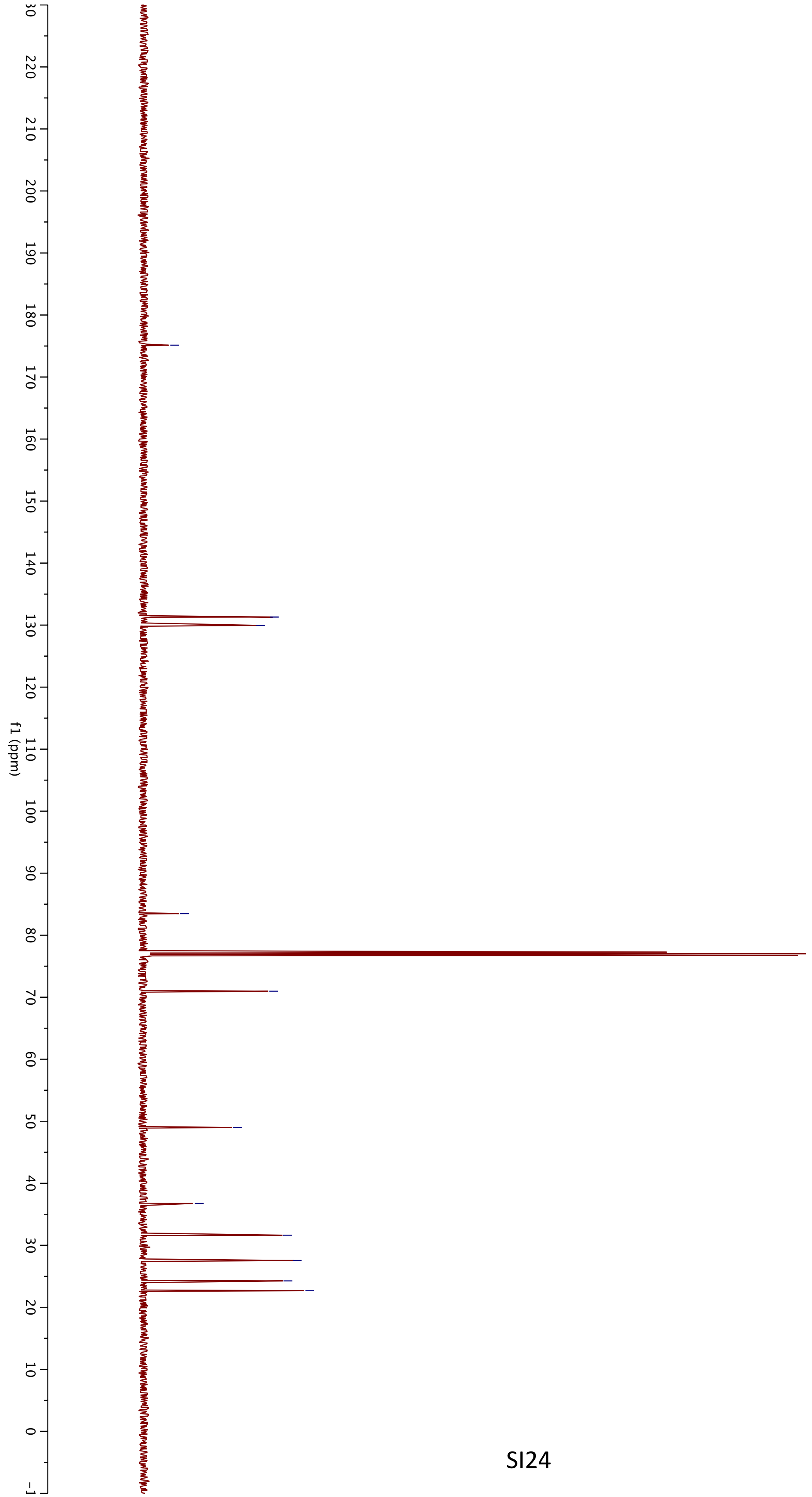

References:

1. A. W. Schuppe; Y. Zhao; Y. Liu; T. R. Newhouse. *J Am Chem Soc* 141 (2019) 9191−9196.
2. S. Yao; M. Johannsen; R. G. Hazell; K. A. Jørgensen. *The Journal of organic chemistry* 63 (1998) 118–121.
